# Ruthenium(II) Polypyridyl Complexes as FRET Donors: Structure- and Sequence-Selective DNA-Binding and Anticancer Properties

**DOI:** 10.1021/jacs.2c11111

**Published:** 2023-01-06

**Authors:** Christopher E. Elgar, Nur Aininie Yusoh, Paul R. Tiley, Natália Kolozsvári, Laura G. Bennett, Amelia Gamble, Emmanuel V. Péan, Matthew L. Davies, Christopher J. Staples, Haslina Ahmad, Martin R. Gill

**Affiliations:** † Department of Chemistry, Faculty of Science and Engineering, Swansea University, Swansea SA2 8PP, U.K.; ‡ UPM-MAKNA Cancer Research Laboratory, Institute of Bioscience, Universiti Putra Malaysia, 43400 Serdang, Selangor, Malaysia; § North West Cancer Research Institute, School of Medical Sciences, Bangor University, Bangor LL57 2DG, U.K.; ∥ SPECIFIC IKC, Materials Science and Engineering, Faculty of Science and Engineering, Swansea University, Swansea SA1 8EN, U.K.; ⊥ Department of Chemistry, Faculty of Science, Universiti Putra Malaysia, 43400 Serdang, Selangor, Malaysia

## Abstract

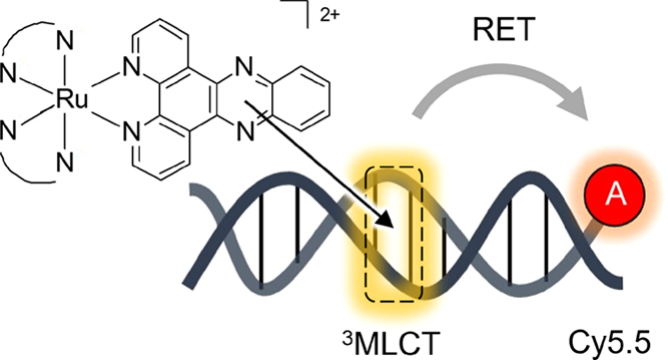

Ruthenium(II) polypyridyl complexes (RPCs) that emit from metal-to-ligand charge transfer (MLCT) states have been developed as DNA probes and are being examined as potential anticancer agents. Here, we report that MLCT-emissive RPCs that bind DNA undergo Förster resonance energy transfer (FRET) with Cy5.5-labeled DNA, forming mega-Stokes shift FRET pairs. Based on this discovery, we developed a simple and rapid FRET binding assay to examine DNA-binding interactions of RPCs with diverse photophysical properties, including non-“light switch” complexes [Ru(dppz)_2_(5,5′dmb)]^2+^ and [Ru(PIP)_2_(5,5′dmb)]^2+^ (dppz = dipyridophenazine, 5,5′dmb = 5,5′-dimethyl-2,2′-bipyridine, PIP = 2-phenyl-imidazo[4,5-*f*][1,10]phenanthroline). Binding affinities toward duplex, G-quadruplex, three-way junction, and mismatch DNA were determined, and derived FRET donor–acceptor proximities provide information on potential binding sites. Molecules characterized by this method demonstrate encouraging anticancer properties, including synergy with the PARP inhibitor Olaparib, and mechanistic studies indicate that [Ru(PIP)_2_(5,5′dmb)]^2+^ acts to block DNA replication fork progression.

## Introduction

Ruthenium(II) polypyridyl complexes (RPCs) that bind DNA by noncovalent mechanisms have been proposed for a multitude of uses in biology, including cellular imaging agents,^1^ anticancer therapeutics,^2^ and photosensitizers for photodynamic therapy.^3^ Based on the discovery that [Ru(bpy)_2_(dppz)]^2+^ (bpy = 2,2′-bipyridine, dppz = dipyrido[3,2-a:2′,3′-*c*]phenazine) binds DNA with high affinity and an accompanying increase in metal-to-ligand charge transfer (^3^MLCT) luminescence—the DNA “light switch” effect^4^—RPCs utilizing intercalating ligands have been developed as high-affinity DNA binders.^5^ Judicious selection or chemical modification of ancillary or intercalating ligands can then generate RPCs that demonstrate site- or structure-selectivity^6−9^ along with tuneable biological activity.^10^ The increase in MLCT emission upon binding presents a convenient method to determine nonspecific binding site affinity via the generation of Scatchard plots that fit the McGhee and von Hippel equation.^11^ However, this equation is unsuitable for oligonucleotides of known sequences, which are vital in determining site- or structure-specific binding,^8,12^ and reliance on MLCT emission also inherently disfavors the study of RPC/DNA combinations that possess variable, weak, or complete absence of “light switch” properties. This latter category of RPCs includes molecules of considerable biological interest such as [Ru(N^∧^N)_2_(PIP)]^2+^ (PIP = 2-phenyl-imidazo[4,5-*f*][1,10]phenanthroline)^13^ and [Ru(dppz)_2_(N^∧^N)]^2+^ complexes.^14^ Although luminescence-independent techniques exist to examine and quantify DNA binding, including ultraviolet–visible (UV–vis) absorption^15^ and isothermal titration calorimetry (ITC),^16^ these methods are low throughput and comparisons of results obtained using different techniques between studies can be challenging. As such, a single assay suitable for a range of DNA structures and RPCs with diverse photophysical properties and compatible with high-throughput screening methods would be advantageous.

Förster resonance energy transfer (FRET) involves dipole–dipole coupling from the excited state of a donor luminophore (D) to the ground state of an acceptor luminophore (A)—a FRET pair.^17^ Due to its inverse-sixth-power distance dependence, FRET provides proximity detection at the nanometer scale.^17^ Biomolecules labeled with FRET pairs are utilized in bioassays to probe the biomacromolecular structure, protein–protein or protein–DNA interactions,^18−20^ including screening for small molecules that inhibit (or promote) these processes,^21^ while inherently fluorescent or fluorescently labeled compounds and target receptors facilitate the development of advanced binding assays.^22,23^ Advantages of FRET are its high sensitivity, broad dynamic range, and compatibility with high-throughput screening.^17^ MLCT-emissive phosphorescent RPCs hold significant potential as FRET donors, where the low energy and large Stokes shifts of MLCT phosphorescence (typically >150 nm) would be advantageous in addressing current limitations arising from the use of two organic fluorophores, which include poor signal-to-noise and spectral bleed through.^24^ Despite this, few studies have examined RPCs in FRET bioassays. Work to date has included a [Ru(bpy)_3_]^2+^-based enzyme-cleavable sensor^25^ and DNA sequences covalently labeled with RPCs to examine DNA–DNA assembly.^26,27^ Studies employing a DNA-binding RPC as a FRET donor are even rarer, yet the potential for this was explored by Lakowicz et al., who demonstrated successful RET between [Ru(bpy)_2_(dppz)]^2+^ and BO-PRO_3_ when both molecules were intercalated to DNA.^28,29^ A disadvantage of this was that two reversibly binding DNA molecules were employed, thereby introducing an additional variable that makes assay development problematic.

**Figure 1:**
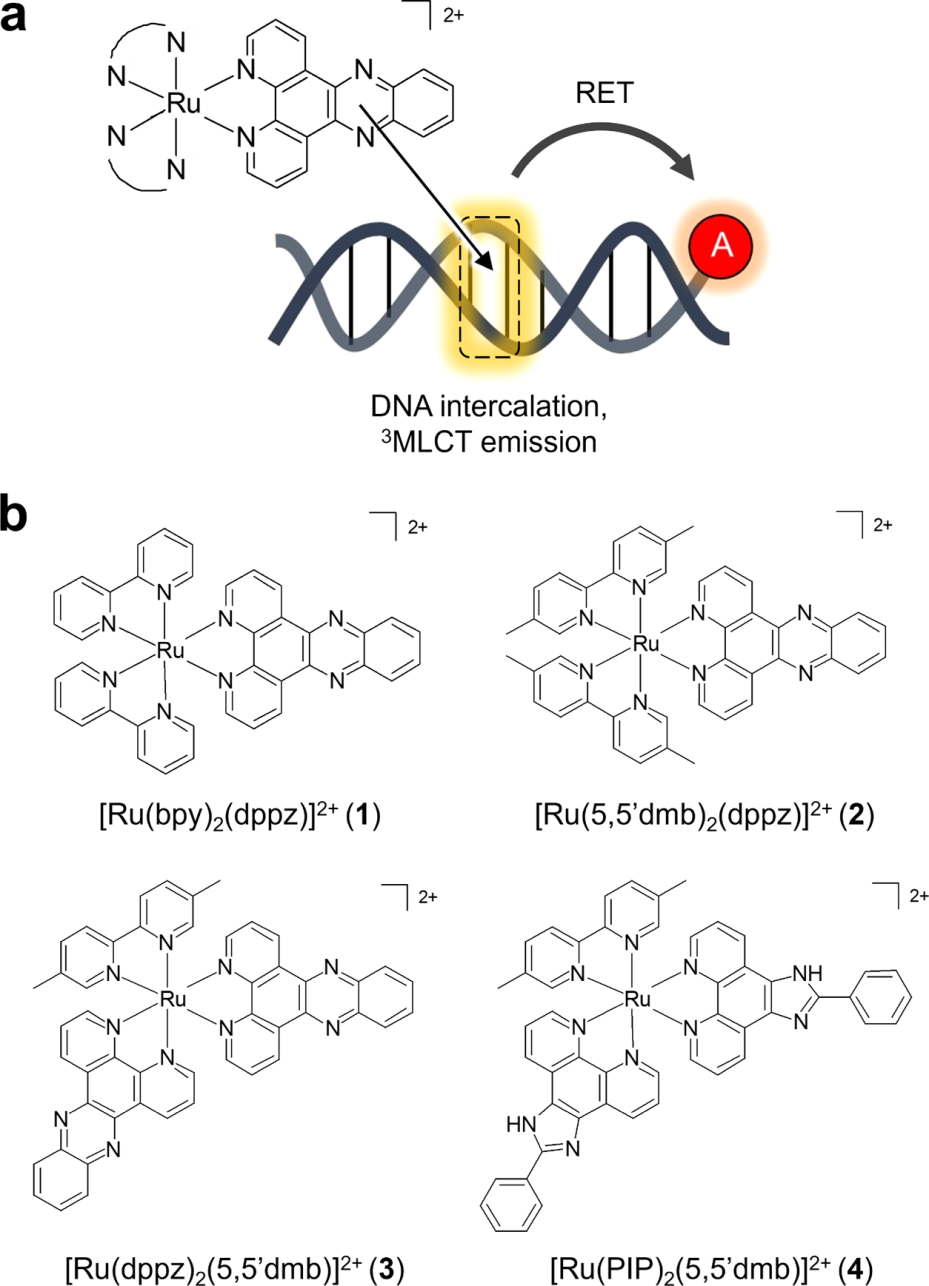
(a) Schematic diagram illustrating DNA-binding ruthenium(II) polypyridyl complexes as FRET donors for fluorophore-labeled DNA. (b) Chemical structures of ruthenium(II) polypyridyl complexes used in this study. Complexes were used as a mixture of stereoisomers.

Here, we report the ability of ^3^MLCT emission from DNA-binding RPCs to undergo resonance energy transfer with a fluorophore covalently linked to DNA (Figure 1a). Using fluorophore-labeled oligonucleotides, we utilize this effect to characterize structure- and sequence-specific DNA binding of four RPCs with diverse photophysical properties, including non-“light switch” molecules, determining affinity—and proximity—of binding in a single, rapid assay. We show that molecules characterized by this method demonstrate encouraging anticancer properties, including impacting DNA replication and synergy with the PARP inhibitor Olaparib in a combination therapy approach.

## Results and Discussion

### Synthesis and Photophysical Properties

[Ru(bpy)_2_(dppz)]^2+^ (**1**) and [Ru(5,5′dmb)_2_(dppz)]^2+^ (5,5′dmb = 5,5′dimethyl bpy) (**2**) were prepared by previously reported methods.^4,8^^1^H NMR, mass spectroscopy, and elemental analysis were in agreement with the published data. The novel complexes [Ru(dppz)_2_(5,5′dmb)]^2+^ (**3**) and [Ru(PIP)_2_(5,5′dmb)]^2+^ (**4**) were prepared in a similar manner via the preparation of the intermediate compounds [Ru(dppz)_2_Cl_2_] and [Ru(PIP)_2_Cl_2_] and characterized by ^1^H NMR, ^13^C NMR, high-resolution mass spectrometry (HRMS), Fourier-transform infrared spectroscopy (FTIR), and elemental analysis. Full synthetic and characterization details are in the Supporting Information (Figures S1–S10). Excitation/emission spectra of the hexafluorophosphate salts of **3** and **4** in acetonitrile demonstrated characteristic ^3^MLCT emission upon ^1^MLCT excitation (Figure S11 and Table S1); however, in aqueous media as their chloride salts, the emission of **3** is almost completely quenched, while **4** retains a relatively high MLCT intensity (Figure S12a). The addition of calf-thymus DNA to each complex in aqueous media reveals a varied photophysical response: **2** acts as a conventional “light switch” molecule, albeit with reduced intensity compared to **1**, **3** does not exhibit “light switch” behavior as only a 2.4-fold enhancement of MLCT emission is seen, and the aqueous MLCT emission of **4** is quenched by the addition of DNA (Table 1 and Figure S12b).

**Table 1:** Photophysical Properties of DNA-Bound **1**–**4** [Donor] and Cy5.5 [Acceptor] FRET Pairs^a^

complex	DNA *k*_b_ (M^–1^)	*n* (base pairs)	λ_ex_ (max) (nm)	λ_em_ (max) (nm)	*I*_(DNA)_/*I*_*(*aq.)_	Φ_(DNA)_	*J* (λ) (M^–1^ cm^–1^·nm^4^)	*R*_o_ (Å)
**1**	2.513 × 10^6^	1.26	450	620	117	0.008	1.50 ± 0.23 × 10^16^	38.9 ± 1.02
**2**	2.632 × 10^6^	1.73	440	635	13	0.004	1.68 ± 0.24 × 10^16^	35.1 ± 0.85
**3**	1.248 × 10^6^	1.14	440	630	2.4	0.0005	1.50 ± 0.08 × 10^16^	38.9 ± 0.34
**4**	nd	nd	470	605	0.56	0.042	1.34 ± 0.16 × 10^16^	50.2 ± 0.97

a Buffer: 5 mM Tris, 200 mM NaCl, pH 7.5. *J* (λ) = spectral overlap integral. *R*_o_ = Förster radius. ND = not determined.

Binding affinities were determined by fitting the McGhee von Hippel binding model^11^ to luminescence titrations, showing that **2** and **3** each bind calf-thymus DNA with binding affinities comparable to **1** (equilibrium binding constants, *K*_b_ of 2.63 × 10^6^ and 1.25 × 10^6^ M^–1^ for **2** and **3**, respectively, Table 1). The binding affinity of **4** could not be determined by either fluorescence or UV–visible absorption titrations; however, apparent binding constants, *K*_app_, from the ethidium bromide displacement assay indicates that **4** possesses a DNA-binding affinity comparable to **3** (Figure S13).

### MLCT-Cy5.5 FRET Pair Characterization

**Figure 2:**
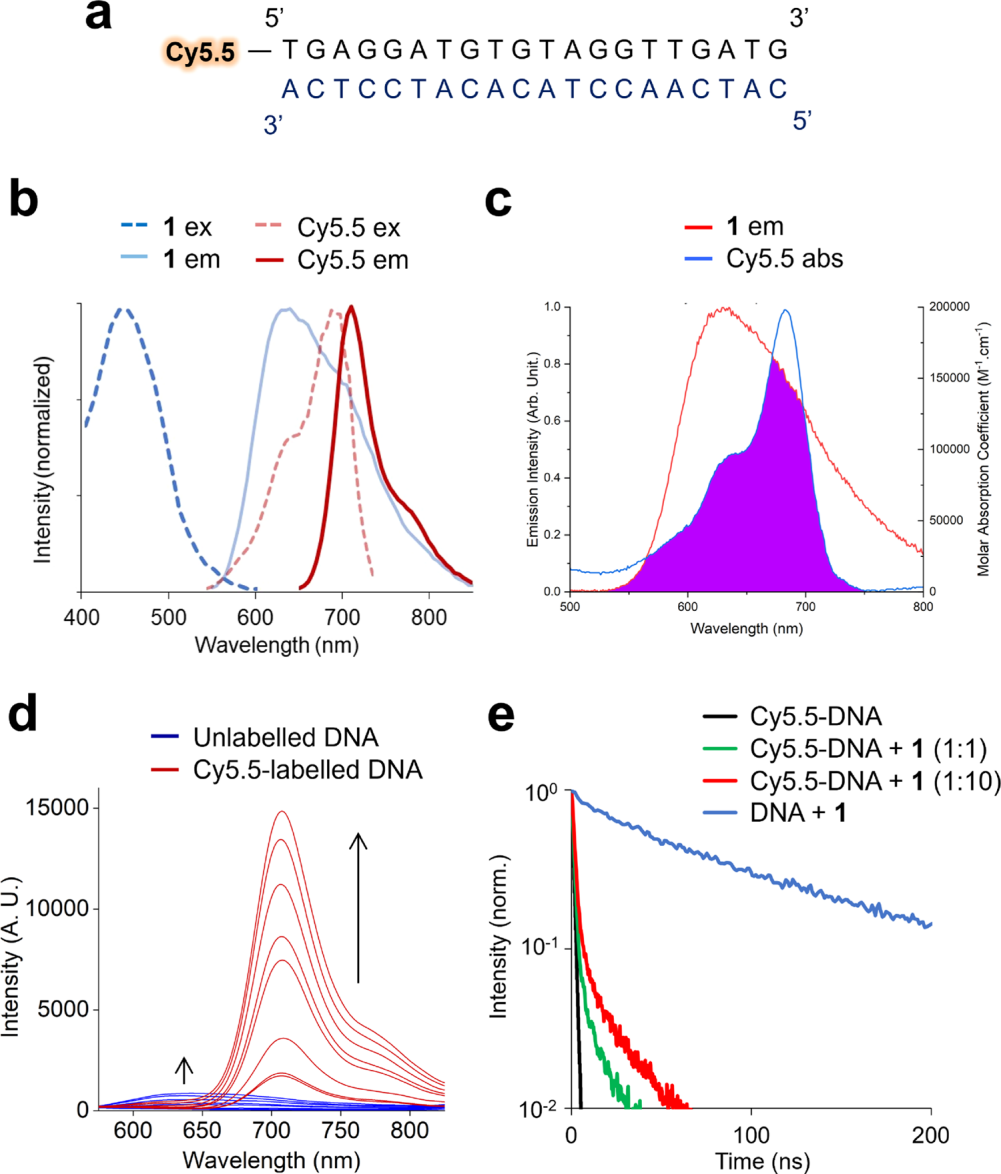
(a) Cy5.5-labeled 20-mer duplex DNA. (b) Excitation (ex, dashed) and emission (em, solid) spectra of DNA-bound RPC [Ru(bpy)_2_(dppz)]^2+^ (blue) and Cy5.5 (red). (c) Spectral overlap of the MLCT emission of DNA-bound **1** and Cy5.5 absorption. (d) Red: the emission spectra (λ_ex_ = 450 nm) of Cy5.5-labeled 20-mer (1 μM) with increasing concentration of **1** (0.1–20 μM). Blue: the addition of **1** to unlabeled DNA showing MLCT emission (λ_ex_ = 450 nm). (e) Lifetime of 710 nm emission in the absence and presence of **1**. Lifetime of unlabeled 20-mer with **1** at 630 nm included for comparison. Buffer: 5 mM Tris, 200 mM NaCl, pH 7.5.

Based on the emission spectra of DNA-bound **1**–**4** (Figure S14 and Table 1), we hypothesized that each RPC would be a suitable donor for the cyanine dye Cy5.5 (λ_ex[max]_ = 683 nm, λ_em[max]_ = 703 nm) to act as the acceptor. Using a Cy5.5-labeled 20-mer duplex (Figure 2a and Table 2), the sufficient spectral overlap of the MLCT emission of DNA-bound **1** and Cy5.5 was demonstrated (Figure 2b,c and Table 1) and the addition of **1** to the Cy5.5-labeled 20-mer followed by 450 nm excitation resulted in an intense emission peak at 710 nm (Figure 2d). These wavelengths correspond to ^1^MLCT excitation of **1** and Cy5.5 emission. Comparing these results to experiments conducted using the unlabeled 20-mer, negligible ^3^MLCT emission at 630 nm was observed for the Cy5.5-labeled 20-mer (Figure 2d), demonstrating successful ^3^MLCT emission quenching in the presence of Cy5.5, consistent with an RET process. In addition to this, a clear increase in the lifetime of the 710 nm Cy5.5 peak in the presence of the donor was observed (Figure 2e), resulting in an average lifetime of 2.4–3.6 ns for the **1**-Cy5.5 FRET pair (average lifetime for Cy5.5-DNA = 1.2 ns, Table S2). Similar results for **2**–**4** were also achieved (Figures S14 and S15 and Table 1), confirming successful RET from the MLCT excited states of the DNA-binding **1**–**4** to Cy5.5-labeled DNA in each case. Förster radii (*R*_0_) were calculated^17^ and found to range from 3.5 nm (**2**-Cy5.5) to 5.0 nm (**4**-Cy5.5) (Table 1). Each new MLCT-Cy5.5 FRET pair has a Stokes shift value of ∼250 nm, some of the largest values described to date, signifying that they classify as “mega-Stokes shift” FRET pairs.^30^

**Table 2:** DNA Sequences Used in this Work

structure	sequences (all written 5′ to 3′)
20-mer duplex	S1: [cyanine55]TGAGGATGTGTAGGTTGATG
	S2: CATCAACCTACACATCCTCA
3WJ	S1: [cyanine55]TGAGGATGTGTAGGTTGATG
	S2: CATCAACCTAAGAATGAGAC
	S3: GTCTCATTCTCACATCCTCA
G-quadruplex	[cyanine55]AGGGTTAGGGTTAGGGTTAGGG
matched duplex	S1: [cyanine55]GACCAGCTTATCACCCCTAGATACCAT
	S2: ATGGTATCTAGGGGTGATAAGCTGGTC
mismatch 1 duplex	S1: [cyanine55]GACCAGCTTATCACCCCTAGATACCAT
	S2: ATGGTATCTAGGGGTGATAAGCTCGTC
mismatch 2 duplex	S1: [cyanine55]GACCAGCTTATCACCCCTAGATACCAT
	S2: ATGGTATCTAGGGCTGATAAGCTGGTC
mismatch 3 duplex	S1: [cyanine55]GACCAGCTTATCACCCCTAGATACCAT
	S2: ATGCTATCTAGGGGTGATAAGCTGGTC

### Structure-Specific DNA Binding

**Figure 3:**
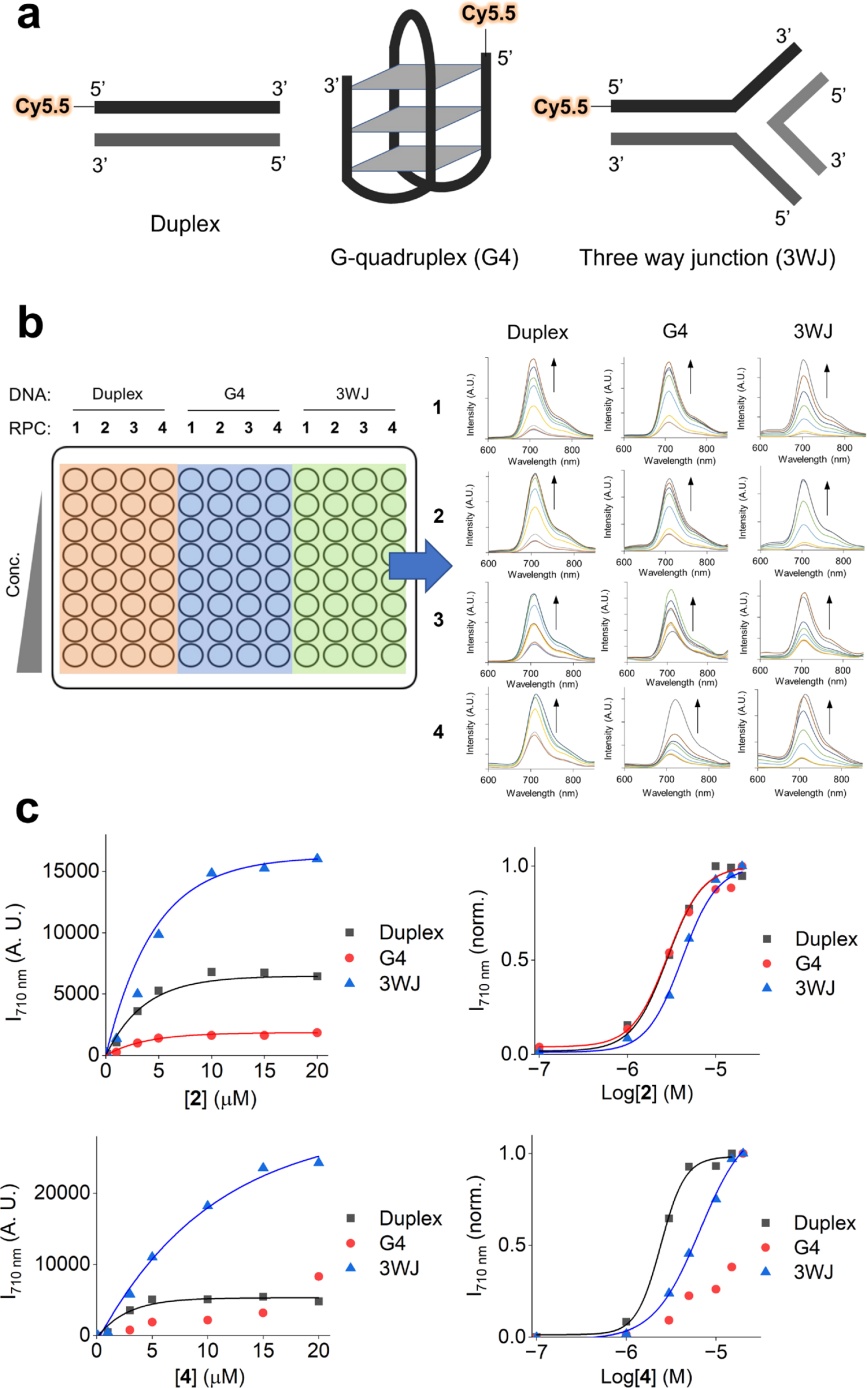
(a) Schematic diagrams of a Cy5.5-labeled duplex, G-quadruplex (G4), and three-way junction (3WJ). (b) Left: an example of a typical experimental setup. Right: the sample emission spectra of Cy5.5-labeled DNA with increasing concentrations of **1**–**4** and successful FRET for each DNA structure/compound combination (λ_ex_ = 450 nm). See Table 2 for precise DNA sequences used to form each structure. (c) FRET intensity (left) and the dose–response curve (right) generated from the addition of **2** or **4** (0.1–20 μM) to Cy5.5-labeled duplex, G4, or 3WJ DNA (1 μM). λ_ex_ = 450 nm, λ_em_ = 710 nm. FRET intensity was background corrected. 3WJ and duplex buffer: 5 mM Tris, 200 mM NaCl, pH 7.5. G4 buffer: 100 mM KCl, 10 mM Tris.HCl, pH 7.4.

Identifying small molecules that bind and stabilize the nonduplex G-quadruplex (G4) and three-way junction (3WJ) structures is a topic of current interest within medicinal chemistry.^31−33^ RPCs are well-established to interact with G-quadruplexes;^34−36^ however, their binding to 3WJs has not been investigated. To examine whether MLCT-Cy5.5 FRET could determine binding affinities of **1**–**4** toward different DNA structures, Cy5.5-labeled oligonucleotides that form a duplex, G-quadruplex (G4),^37^ or a three-way junction (3WJ)^38^ (Figure 3a and Table 2) were treated with a concentration gradient of each complex and the resultant FRET intensity was determined. A 96-well plate and plate reader format was employed to improve throughput, allowing all 12 RPC/DNA combinations to be examined in a single experiment (Figure 3b). Resultant FRET binding curves were fit to a dose–response model to allow derivation of equilibrium dissociation constants, *K*_d_ (Figures 3c and S16 and Table 3), an established means of quantifying small molecule–nucleic acid binding in high-throughput screening.^39^

**Table 3:** Dissociation Constants (*K*_d_) for Binding of **1**–**4** to 20-mer Duplex, G-Quadruplex (G4), and Three-Way Junction (3WJ) DNA Measured Using Cy5.5-Labeled Oligonucleotides and FRET^a^

	duplex		G4		3WJ	
complex	*K*_d_ (μM)	*r* (Å)	*K*_d_ (μM)	*r* (Å)	*K*_d_ (μM)	*r* (Å)
**1**	2.8 ± 0.9	33.5	3.9 ± 1.3	26.8	4.3 ± 0.9	37.4
**2**	2.3 ± 0.5	33.1	3.8 ± 1.7	30.0	4.4 ± 0.7	35.0
**3**	6.3 ± 0.3	23.8	nd	nd	nd	nd
**4**	2.6 ± 1.0	55.9	nd	nd	6.4 ± 0.3	49.6

a See Table 2 for precise DNA sequences used. ND = Not determined (i.e., *K*_d_ values >20 μM). Mean +/– SD of two independent experiments.

Low micromolar *K*_d_ values for **1**–**4** toward the 20-mer duplex compare favorably with known small molecule DNA aptamer affinities^40^ and provide a binding affinity order of **2** > **4** > **1** > **3** (Table 3). In addition to duplex DNA, both **1** and **2** showed high affinity toward G4 (Table 3) and the comparable binding affinities for **1** toward duplex and quadruplex DNA are in agreement with other works.^35^ In contrast to **1** and **2**, however, neither **3** nor **4** reached binding saturation with G4 in the concentration range tested, indicating reduced affinity toward G4 for these two complexes. This may be explained by the greater steric bulk of each molecule as **3** and **4** each possess two intercalating ligands coordinated to an octahedral Ru(II) center and is consistent with the observation that the majority of quadruplex-binding small molecules are planar, typically interacting with G4 by G-tetrad stacking.^32^ In addition to duplex and G4 DNA, the FRET binding assay provided a clear indication of the binding of each complex with 3WJ with a binding order of **1** > **2** > **4** > **3** (Figures 3c and S16 and Table 3). All complexes displayed a reduced affinity for 3WJ compared to the duplex; however, this may be rationalized by the greater number of duplex binding sites of the 3WJ structure.

As FRET can also determine donor–acceptor proximity at the nanometer scale,^17^ mean D–A (*r*) distances were calculated. In our FRET assay, these values correspond to the average distance between the DNA-bound RPC (D) and Cy5.5 label (A). As the location of the Cy5.5 label in the DNA structure is known, this can provide an indication of potential binding sites of the RPC. D–A distances calculated in this manner were found to range from 2.4 to 5.6 nm (Table 3), values consistent with the size of DNA structures employed.^41,42^

### Development of a Mismatch Detection Assay

**Figure 4:**
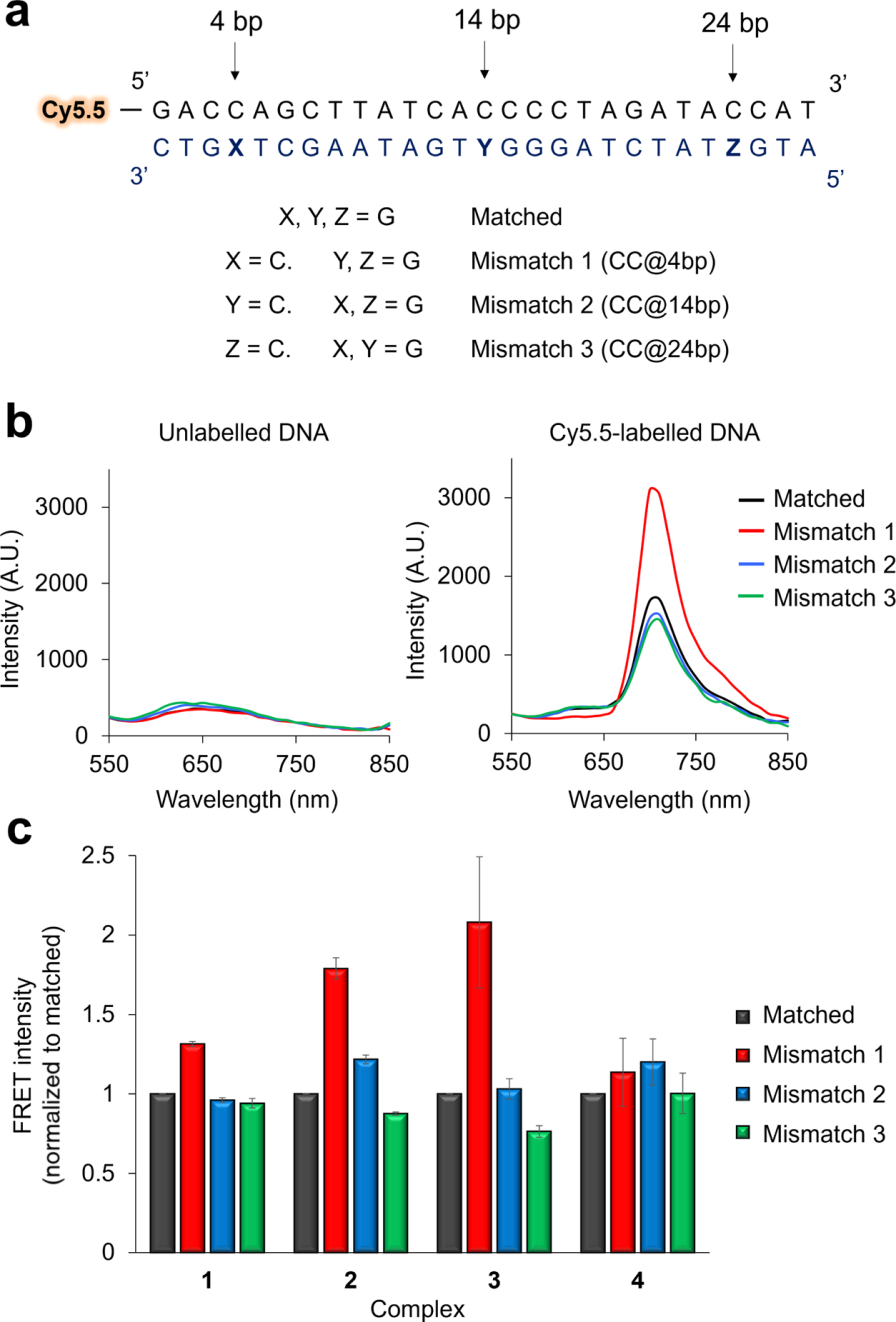
(a) Matched and mismatch-containing Cy5.5-labeled 27-mer DNA duplexes. (b) Emission spectra of **3** (5 μM) and unlabeled (left) or Cy5.5-labeled (right) 27-mers. 1 μM DNA was employed in each case. λ_ex_ = 450 nm, all emission spectra were collected using the same optical parameters. (c) FRET intensity of **1**–**4** (3 μM) with the addition of Cy5.5-labeled 27-mer duplexes (1 μM). λ_ex_ = 450 nm and λ_em_ = 710 nm. FRET intensity was normalized to matched DNA. Data are average of two independent repeats +/– SD. Buffer: 5 mM Tris, 200 mM NaCl, pH 7.5.

In addition to structure selectivity, RPCs can exhibit sequence-specific binding, such as toward duplexes that contain non-Watson Crick mismatched base pairs (mismatches).^7−9,43^ Accordingly, the interaction of **1**–**4** with Cy5.5-labeled 27-mer duplexes containing a CC mismatch was examined by the FRET binding assay (Figure 4a). Duplexes were designed so that the mismatch was located at differing proximities from the Cy5.5 label: 4, 14, or 24 base pairs. FRET intensity and *K*_d_ values for each duplex/complex combination were determined in a similar manner as described previously and preferential mismatch binding was assessed by comparison to the well-matched control sequence. Employing this methodology, it was apparent that **3** displays the greatest FRET intensity with the addition of the “Mismatch 1” duplex that contains the CC mismatch at the 4 bp position (Figure 4b,c). Furthermore, when the CC mismatch is “moved” further from the Cy5.5 label in the case of the “Mismatch 2” and “Mismatch 3” duplexes, FRET intensity decreases. As the MLCT emission intensity of **3** remains constant with the addition of all four unlabeled 27-mers (Figures 4b and S17b), this confirms that the observed decrease in FRET intensity for the labeled sequences is the result of the CC mismatch being moved further away from the Cy5.5 label. *K*_d_ values show **3** has a higher binding affinity for the Mismatch 1 duplex compared to all other duplexes tested (Table 4 and Figure S18) and, perhaps most remarkably, D–A distances reveal that **3** is bound in closer proximity to Cy5.5 for this sequence, which contains the CC mismatch closest to the fluorescent label (Table 4). Similar behavior was also observed for **2**, a molecule that has been previously reported to interact with CC mismatches,^8^ and relative FRET intensity was again found to be independent of MLCT emission intensity (Figures 4c and S17). Taken together, these results are consistent with **2** and **3** targeting with the CC mismatch and illustrate how MLCT-Cy5.5 FRET can be employed to identify mismatch interactive RPCs, including the non-“light switch” complex **3**. It is also noteworthy that all complexes displayed reduced binding affinities toward the matched 27-mer compared to the 20-mer duplex above. This may be rationalized by the increased number of binding sites presented by the larger duplex requiring a greater concentration of each complex for binding saturation and this observation illustrates that internal controls of comparable DNA sizes are required for the interpretation of binding constant data.

**Table 4:** Dissociation Constants (*K*_d_) and Mean Donor–Acceptor Distances (*r*) for the Binding of **1**–**4** to Cy5.5-Labeled 27-mers Measured Using FRET^a^

	matched		mismatch 1		mismatch 2		mismatch 3	
Complex	K_d_ (μM)	r (Å)	K_d_ (μM)	r (Å)	K_d_ (μM)	r (Å)	K_d_ (μM)	r (Å)
**1**	5.1 ± 0.4	47.6	5.0 ± 0.7	42.2	5.3 ± 0.6	45.4	6.5 ± 1.8	49.7
**2**	4.7 ± 0.1	47.7	4.6 ± 0.6	38.5	4.3 ± 0.6	42.9	5.1 ± 0.3	45.4
**3**	8.1 ± 1.7	54.7	6.4 ± 1.7	44.1	8.3 ± 3.0	56.3	8.1 ± 3.7	49.6
**4**	7.9 ± 2.5	49.9	7.3 ± 2.5	42.4	nd	nd	7.6 ± 1.9	52.5

a ND = not determined (i.e., *K*_d_ > 20 μM). Mean +/– SD of two or three independent experiments.

### Cytotoxicity and Impact on DNA Replication

RPCs that bind DNA by intercalation can stall DNA replication forks, inhibiting cell proliferation,^44,45^ and mismatch binding complexes have shown increased cytotoxicity toward mismatch-mediated repair (MMR)-deficient cancer cell lines.^8,43^ As our FRET binding studies show that **2**–**4** bind DNA with moderate affinity and also indicate that **2** and **3** interact with mismatches, their cytotoxic properties were examined in a small panel of cancer cell lines, including MMR-deficient HCT116 colorectal carcinoma cells. The low bioactivity of **1** has been reported elsewhere^46^ and so was not included in these studies. Assessing the impact on cell viability by the MTT assay, **3** and **4** each display moderate cytotoxicity toward the four cancer cell lines, particularly evident in HCC38 human triple-negative breast cancer (TNBC) cells (derived half inhibitory IC_50_ values of 3.8 and 16.5 μM, respectively, Figure S19 and Table 5). No clear evidence of enhanced cytotoxicity toward MMR-deficient HCT116 cells was observed for **2** or **3** and, considering that **3** also demonstrates a comparable cytotoxicity profile to the nonmismatch interactive **4**, these results suggest cytotoxicity occurs through a mismatch-independent pathway. In comparison to their anticancer activity, both **3** and **4** showed significantly reduced effects toward normal MCF10A human breast epithelial cells (IC_50_s >100 and 63.1 μM for **3** and **4**, respectively), indicating that both complexes demonstrate greater potency and enhanced cancer selectivity than cisplatin in MDA-MB-231 TNBC cells (Figure S19 and Tables 5 and S3).

**Table 5:** Half Inhibitory (IC_50_) Values of **2**, **3**, or **4** in MDA-MB-231 or HCC38 Breast Cancer, HCT116 Colorectal Carcinoma, T24 Bladder Cancer Cell Lines, or the MCF10A Non-Tumorigenic Epithelial Cell Line (72 h Treatment)^a^

complex	MDA-MB-231	HCC38	HCT116	T24	MCF10A
cisplatin	32.5 ± 11.7	4.5 ± 0.3	5.0 ± 1.4	1.5 ± 0.5	26.5 ± 8.2
**2**	>100	>100	53.7 ± 9.9	34.4 ± 3.3	>100
**3**	21.8 ± 2.9	3.8 ± 2.5	16.4 ± 1.4	28.0 ± 4.4	>100
**4**	20.5 ± 9.6	16.5 ± 4.2	9.9 ± 1.8	29.9 ± 2.3	63.1 ± 21.0

a Cisplatin treatment is included for comparison. Data are mean ± SD of three independent experiments.

**Figure 5:**
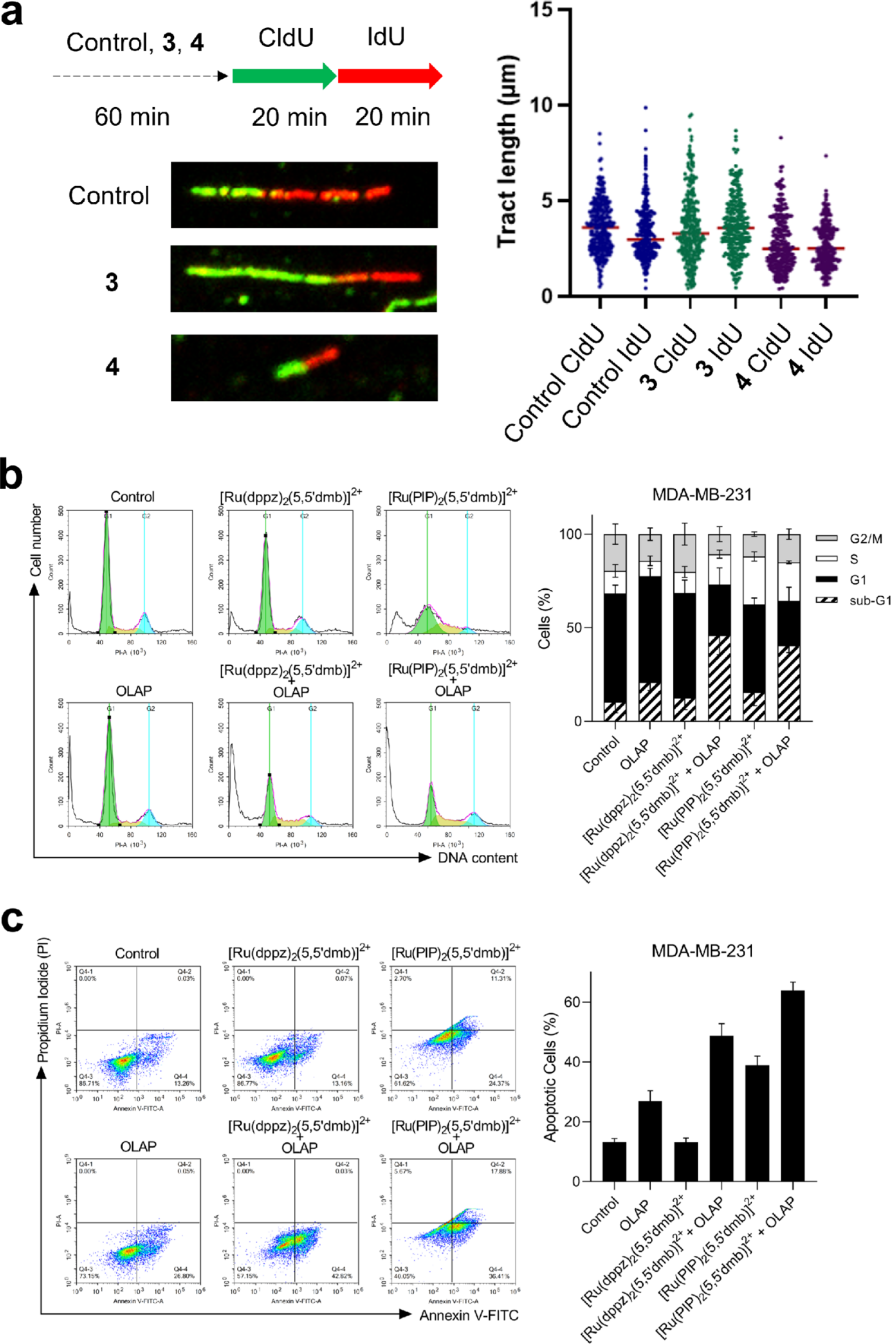
(a) Left: a DNA fiber assay scheme. MDA-MB-231 cells were treated with the DMSO control, **3** or **4** (20 μM, 1 h), then pulse-labeled with CldU followed by IdU for 30 mins each. Examples of fiber images after immunofluorescence are shown. Right: the quantification of CldU and IdU tract lengths from at least 150 fibers per experimental condition, two independent repeats. (b) Left: the representative cell-cycle plots of MDA-MB-231 cells treated with the stated single-agent (10 μM) alone or in combination with Olaparib (10 μM) for 72 h, as determined by flow cytometry. Right: the quantification of cell-cycle phase distributions. Data were expressed as mean ± SD of two independent experiments (*n* = 2). (c) Left: the annexin V-FITC assay and representative dot plots of MDA-MB-231 cells treated with the stated single-agent (10 μM) alone or in combination with Olaparib (10 μM) for 72 h. Right: the quantification of apoptotic (annexin V positive) cells. Data were expressed as mean ± SD of two independent experiments (*n* = 2).

To examine the impact of **3** and **4** on DNA replication, a DNA fiber assay on treated cells followed by sequential CldU and IdU labeling was performed.^47^ In this manner, treating MDA-MB-231 cells with **4** for 1 h results in a marked decrease in both CldU and IdU tract lengths compared to control cells (Figure 5a), thereby providing direct evidence that **4** acts to decrease DNA replication fork speed and interferes with DNA replication. Furthermore, treatment with **4** resulted in substantial disruption to cell-cycle progression, with an increase in S-phase cells and an accompanying decrease in the G2/M phase compared to the control (Figure 5b). Examining cell fate, an increase in cells with sub-G1 DNA content and annexin V positive population indicate the induction of apoptosis by **4** (Figure 5b,c). These results are consistent with **4** inhibiting DNA replication, inducing S-phase arrest, and ultimately triggering cell death by apoptosis. Interestingly, and despite possessing comparable DNA-binding affinities and IC_50_ concentrations to **4**, **3** did not impact DNA replication nor cell-cycle progression in a similar manner, a finding that implies the PIP ligand is required for the DNA replication inhibition shown by **4**.

### Synergy with PARP Inhibitor Olaparib

Finally, RPCs that target DNA can achieve synergistic cancer cell killing with the PARP inhibitor Olaparib, a strategy that can enhance both cytotoxicity and cancer selectivity.^48^ Cotreatment of the panel of cancer cell lines with **2**, **3**, or **4** alongside a low dose (10 μM) of the PARP inhibitor Olaparib resulted in enhanced cytotoxicity of each complex compared to single-agent conditions (Figures S19 and S20). Derived IC_50_ concentrations and combination indices (CIs) provided evidence of a synergistic relationship between the two RPCs and Olaparib in MDA-MB-231 cells (Table S4 and Figure S21). Briefly examining the mechanism of synergy, MDA-MB-231 cells treated with **3** or **4** alongside Olaparib showed an increase in sub-G1 content and annexin V positive cells compared to single-agent conditions (Figure 5b,c). These results indicate elevated apoptosis as a result of each combination in a similar manner to previous work utilizing the structurally-related PARP hypersensitizer [Ru(dppz)_2_(PIP)]^2+^.^49^ That **3** and **4** demonstrate evidence of PARPi synergy is consistent with both molecules inducing DNA damage; however, when combined with the results above, this implies distinct mechanistic differences between the two complexes: **4** most likely achieves PARPi synergy via stalling DNA replication forks while **3** operates by an as yet unknown mechanism of DNA damage generation. Future work will explore this in more detail.

## Conclusions

In conclusion, we have developed a FRET-based method that can be employed to examine the ability of RPCs to bind Cy5.5-labeled DNA. We show that MLCT-Cy5.5 FRET is compatible with DNA-binding RPCs with a range of MLCT-emissive properties and numerous DNA structures and sequences, where it may be used to quantify structure- and sequence-specific binding together in a single rapid assay. The advantages of MLCT-Cy5.5 FRET identified include a high signal-to-noise ratio, wavelength shifting and intensity enhancement of “light switch” emission, and the generation of a DNA-dependent FRET peak for non-“light switch” complexes. Furthermore, as the binding molecule is the FRET donor and the target DNA is the FRET acceptor, the derived proximity of the binding FRET pair provides an indication of the binding site. Employing this method, we identify a new mismatch-interactive RPC and show that molecules characterized by this method possess encouraging anticancer activity, including evidence of DNA replication inhibition and synergy with the PARP inhibitor Olaparib.

## Supporting Information Available

Experimental section; characterization data for **3** and **4**; photophysical properties of **3** and **4** as PF_6_- salts in acetonitrile; ethidium bromide displacement assay; emission spectra for compounds with DNA; additional dose-response curves; cell viability data; selectivity indices; IC_50_ values of **2**, **3**, or **4** in combination with Olaparib. (PDF)
