# Supplementary material for: Ruthenium(II) Polypyridyl Complexes as FRET Donors: Structure- and Sequence-Selective DNA-Binding and Anticancer Properties
Source: J Am Chem Soc. 2023 Jan 6;145(2):1236–46. doi: 10.1021/jacs.2c11111 (PMC9853847; doi:10.1021/jacs.2c11111)
Supplement: Supplementary file 1 — ja2c11111_si_001.pdf [file ja2c11111_si_001.pdf]

Supporting Information for:

Ruthenium(II) polypyridyl complexes as FRET donors: Structure- and sequence-selective DNA binding and anti-cancer properties

Christopher E. Elgar,[a] Nur Aininie Yusoh,[b] Paul R. Tiley,[a] Natália Kolozsvári,[a] Laura G. Bennett,[c] Amelia Gamble,[c] Emmanuel V. Pean,[d] Matthew L. Davies,[d] Christopher J. Staples,[c] Haslina Ahmad[e] and Martin R. Gill[a*]

a Department of Chemistry, Faculty of Science and Engineering, Swansea University, Swansea, SA2 8PP, UK

b UPM-MAKNA Cancer Research Laboratory, Institute of Bioscience, Universiti Putra Malaysia, 43400 Serdang, Selangor, Malaysia

c North West Cancer Research Institute, School of Medical Sciences, Bangor University, Bangor, LL57 2DG, UK

d SPECIFIC IKC, Materials Science and Engineering, Faculty of Science and Engineering, Swansea University, Swansea, SA1 8EN, UK

e Department of Chemistry, Faculty of Science, Universiti Putra Malaysia, 43400 Serdang, Selangor, Malaysia

m.r.gill@swansea.ac.uk

Experimental Section p2

Supplementary Tables p8

Supplementary Figures p10

References p27

Experimental Section

General

All chemical reagents and solvents were purchased from commercial sources (Sigma-Aldrich, ThermoScientific) and used as supplied. ^{1}H and ^{13}C NMR spectra were obtained using a Bruker Advance III 500 MHz Nuclear Magnetic Resonance Spectrometer. HRMS (high resolution mass spectroscopy) samples were analysed at the EPSRC UK National Mass Spectrometry Facility at Swansea University using a ThermoScientific LTQ Orbitrap XL 1 Mass Spectrometer. Fourier Transform Infrared Spectra were run on a Perkin Elmer FT-IR Spectrometer Spectrum TWO. Elemental analysis was performed by the Elemental Analysis Service at London Metropolitan University.

Complex synthesis and characterisation

$[Ru(N^\wedge N)_2Cl_2]$ intermediate complexes were prepared using a modified method described by Sullivan et al.[1] Briefly, $RuCl_3.3H_2O$ was added to a stirring solution of ligand (2 eq) in DMF. LiCl (7 eq) was added, and the mixture was sparged with N_2 for 20 minutes and protected from light. The mixture was then heated at reflux for 8 hours under nitrogen atmosphere. The products were precipitated from solution by the addition of acetone and collected by filtration and washed with distilled water and diethyl ether. The resultant brown intermediates were used in subsequent reactions without further purification or characterisation. $[Ru(bpy)_2(dppz)]^{2+}$ (**1**) and $[Ru(5,5'dmb)_2(dppz)]^{2+}$ (**2**) were prepared by previously-reported methods[2,3] from $[Ru(bpy)_2Cl_2]$ and $[Ru(5,5'dmb)_2Cl_2]$, respectively.

$[Ru(bpy)_2(dppz)]^{2+}$ (**1**). ^{1}H NMR (500 MHz, d_6-acetone), δ, ppm: 9.78 (d, J = 1.2 Hz, 2H), 8.88 (s, 2H), 8.85 (s, 2H), 8.56 (m, 2H), 8.52 (m, 2H), 8.29 (td, J = 8.0, 1.4 Hz, 4H), 8.20 (d, J = 5.0 Hz, 4H), 8.12 (d, J = 5.4 Hz, 4H), 7.66 (dd, J = 4.2, 2.7 Hz, 2H), 7.42 (d, J = 5.7 Hz, 2H). HRMS for $RuC_{38}H_{26}N_8Cl_2$: $[M-2Cl]^{2+}$ at 348.0660. FTIR: 555, 831, 1422 and 1448 cm^{-1}.

$[Ru(5,5'dmb)_2(dppz)]^{2+}$ (**2**)**.** ^{1}H NMR (500 MHz, d_6-acetone), δ, ppm: 9.74 (d, J = 1.1 Hz, 2H), 8.70 (d, J = 8.4 Hz, 2H), 8.65 (s, 2H), 8.53 (d, J = 3.0 Hz, 2H), 8.51 (m, 2H), 8.22 (dd, J = 6.5, 3.4 Hz, 2H), 8.09 (d, J = 5.4 Hz, 2H), 8.07 (s, 2H), 7.97 (s, 2H), 7.87 (s, 2H), 7.20 (dd, J = 28.4, 7.4 Hz, 2H), 2.27 (s, 6H), 2.01 (s, 6H). HRMS for $RuC_{42}H_{34}N_8Cl_2$: $[M-2Cl]^{2+}$ at 376.0972. FTIR: 556, 833 and 1474 cm^{-1}.

$[Ru(dppz)_2(5,5'dmb)]^{2+}$ (**3**): $[Ru(dppz)_2Cl_2]$ (100 mg, 136 mmol) and 5,5'-dimethyl-2,2'-bipyridine (30 mg, 163 mmol) were dissolved into ethylene glycol, before the solution was sparged with nitrogen for 20 minutes. The mixture was heated at reflux for 6 hours under a nitrogen atmosphere in the absence of light. Concentrated aqueous $[NH_4][PF_6]$ was added once the solution had cooled to room temperature, and the precipitate collected by filtration, before washing with distilled water and diethyl ether. The crude complex was purified by column chromatography using neutral alumina, collecting the first

reddish orange band eluted using a 1:1 Mixture of acetonitrile and toluene. The solvent was removed *in vacuo* to yield an orange powder (85 mg, 58 %).

$[Ru(dppz)_2(5,5'dmb)]^{2+}$ (**3**) ^{1}H NMR (500 MHz, d_6-acetone) δ 9.81 (dd, J = 8.3, 1.0 Hz, 2H), 9.70 (dd, J = 8.2, 1.0 Hz, 2H), 8.74 (d, J = 8.4 Hz, 2H), 8.67 (dd, J = 5.3, 1.0 Hz, 2H), 8.56 – 8.48 (m, 6H), 8.24 – 8.18 (m, 4H), 8.16 (dd, J = 8.3, 5.4 Hz, 2H), 8.07 (d, J = 3.8 Hz, 2H), 8.04 (d, J = 8.5 Hz, 2H), 7.91 (dd, J = 8.2, 5.4 Hz, 2H), 2.10 (s, 6H) ppm. ^{13}C NMR (126 MHz, d_6-acetone) δ 155.9, 155.3, 155.1, 153.2, 152.0, 151.8, 143.6, 141.1, 139.8, 139.3, 134.6, 134.6, 133.5, 131.8, 131.7, 130.6, 130.5, 128.5, 128.4, 124.4, 29.8, 18.4 ppm. UV-vis (MeCN): λ_{abs} (ε / 10^4 L mol^{-1} cm^{-1}) 204 (7.5), 279 (12.3), 314 (3.5), 457 (2.8), 366 (2.7), 426 (1.6), 460 (1.6) nm. FTIR (solid) (ATR) v_{max}: 3084 (sp^2 C-H), 1477 (Ar C=C), 1421 (Ar C=C), 835 (PF_6), 813, 763, 729, 556 (PF_6) cm^{-1}. HRMS (ESI) m/z: $[M – PF_6]^+$ Calc'd for $C_{48}H_{32}N_{10}RuPF_6$ 995.1499; Found 995.1501. $[M – 2PF_6]^{2+}$ Calc'd for $C_{48}H_{32}N_{10}Ru$ 425.0927; Found 425.0920. Elemental analysis for [**3**]$(PF_6)_2.H_2O$, $C_{48}H_{34}N_{10}RuP_2F_{12}O$: Calc'd: C; 49.8, H; 3.0; N 12.1. Found: C; 48.1, H; 2.9, N; 11.02.

$[Ru(PIP)_2(5,5'dmb)]^{2+}$ (**4**): $[Ru(PIP)_2Cl_2]$ (100 mg, 131 mmol) and 5,5'-dimethyl-2,2'-bipyridine (29 mg, 157 mmol) were dissolved into ethylene glycol, before the solution was sparged with nitrogen for 20 minutes. The mixture was heated at reflux for 6 hours under a nitrogen atmosphere in the absence of light. Concentrated aqueous $[NH_4][PF_6]$ was added once the solution had cooled to room temperature, and the precipitate collected by filtration, before washing with distilled water and diethyl ether. The crude complex was purified by column chromatography using neutral alumina, collecting the first reddish orange band eluted using a 1:1 Mixture of acetonitrile and toluene. The solvent was removed *in vacuo* to yield an orange powder (19.4 mg, 13 %).

$[Ru(PIP)_2(5,5'dmb)]^{2+}$ (**4**) ^{1}H NMR (500 MHz, d_6-acetone) δ 9.22 (d, J = 8.2 Hz, 2H), 9.08 (d, J = 8.1 Hz, 2H), 8.72 (d, J = 8.4 Hz, 2H), 8.46 (d, J = 5.0 Hz, 2H), 8.40 – 8.31 (m, 6H), 8.17 (d, J = 5.2 Hz, 2H), 8.04 – 7.98 (m, 4H), 7.95 (s, 2H), 7.61 (dd, J = 13.8, 7.3 Hz, 8H), 2.10 (s, 6H) ppm. ^{13}C NMR (126 MHz, d_6-acetone) δ 155.9, 153.9, 152.9, 151.4, 151.3, 147.1, 146.8, 139.5, 139.2, 131.4, 131., 130.4, 130.1, 127.5, 127.0, 127.0, 124.2, 18.3 ppm. UV-vis (MeCN): λ_{abs} (ε / 10^4 L mol^{-1} cm^{-1}) 217 (6.7), 251 (4.8), 298 (12.9), 336 (6.8), 343 (6.6), 424 (1.6), 473 (1.7), 509 (1.1) nm. FTIR (solid) (ATR) v_{max}: 3378 (N-H), 3082 (sp2 C-H), 1604 (C=N), 1477 (Ar C=C), 1457 (Ar C=C), 1362 (C-N), 835 (PF_6), 780, 742, 723, 690, 556 (PF_6) cm^{-1}. HRMS (ESI) m/z: $[M – PF_6]^+$ Calc'd for $C_{50}H_{36}N_{10}RuPF_6$ 1023.1809; Found 1023.1807, $[M – 2PF_6]^{2+}$ Calc'd for $C_{50}H_{36}N_{10}Ru$ 439.1084; Found 439.1075. Elemental analysis for [**4**]$(PF_6)_2.3H_2O$, $C_{50}H_{42}N_{10}RuP_2F_{12}O_3$: Calc'd: C; 49.1, H; 3.5, N; 11.5. Found: C; 47.2, H; 3.2, N; 10.7.

For biological studies, complexes were converted to their chloride salts by anion metathesis. 2 mM stock solutions of **1-3** were prepared in dd H_2O, while a 2 mM stock solution of **4** was prepared in 10 % DMSO, 90 % dd H_2O.

Calf thymus DNA binding

Calf thymus DNA was dissolved in Tris buffer and sonicated for 2 x 15 mins. Absorbances at 260 nm and at 280 nm were measured using UV/VIS spectrometer and $A_{260\,nm}/A_{280\,nm} > 1.8$ indicated a protein-free sample. The concentration of the CT DNA solution was determined by $A_{260\,nm}/\varepsilon_{260\,nm}$, where $\varepsilon_{260\,nm} = 6600\ dm^3\ mol^{-1}\ cm^{-1}$. Luminescence titrations were performed by adding increasing aliquots of a stock DNA solution of known concentration to a cuvette containing **1-4.** After each aliquot addition, solutions were allowed to equilibrate for 2 minutes and luminescence emission spectra were recorded. At least 20 data points before the emission intensity reached a maximum were obtained. Derived curves showing the fraction of complex bound to DNA versus [DNA]/[complex] were used to generate Scatchard plots and data were fit the McGhee-von Hippel equation[4] to determine binding constants K_b and site sizes (number of base pairs per site), n, as described in related publications.[5,6]

Ethidium bromide displacement assay

Based on a method optimized by McCann et al.,[7] a working solution of 20 µM CT DNA and 25.2 µM EthBr in Tris buffer was prepared and allowed to equilibrate for 2 hrs. This solution was mixed 1:1 with a concentration gradient of each compound in a 96 well plate to give final concentrations of 10 µM CT DNA, 12.1 µM EthBr and the concentration gradient of **1-4** required. After mixing, solutions were allowed 1 h to equilibrate and ethidium bromide fluorescence measured by plate reader ($\lambda_{ex} = 530$ nm, $\lambda_{em} = 610$ nm). Due to spectral bleed-through, the results for **4** were adjusted for the emission of the ruthenium(II) complex by subtracting non-ethidium bromide containing comparison values at these excitation/emission wavelengths. Data were expressed as a decrease in ethidium bromide fluorescence compared to the maximum intensity in the absence of compound. Apparent binding constants, K_{app}, were calculated from Equation 1, where $k_{EB} = 9.5 \times 10^6\ M^{-1}$ and C_{50} = concentration of complex required to reduce emission intensity by 50%.

$$k_{app} = \frac{k_{EB} \times [EB]}{C_{50}} \qquad \textit{Equation 1}$$

DNA preparation from oligonucleotides

Unlabeled and Cy5.5-labeled oligonucleotides were purchased from Merck (HPLC purification standard) and used as supplied. Oligonucleotides were mixed together for the required DNA structure, heated to 95 °C over a 10 minute period, then gradually cooled and stored overnight at 4 °C before use. Duplexes and 3WJ were prepared in 5 mM Tris, 200 mM NaCl, pH 7.5. G-quadruplex DNA was

prepared as described in Waller et al. employing a 100 mM KCl and 10 mM Tris.HCl buffer at pH 7.4.[8] The three oligonucleotides that form the 3WJ structure were selected and prepared as described in Zhu et al.[9]

Photophysical characterisation

Absorption spectra were recorded on a UV/VIS Lambda 365 Spectrometer (Perkin Elmer) and fluorescence spectra were run on an LS 55 Fluorescence Spectrometer (Perkin Elmer) or plate reader (Tecan Infinite M Nano). Lifetime measurements were recorded on a Lifespec 2 (Edinburgh Instruments) equipped with an EPL405 or EPL635 laser.

Luminescence quantum yields of **1-4** were calculated by Equation 2, where Φ_D is quantum yield, m is the gradient of luminescence versus absorbance, η is a solvent refractive index. Subscripts D and std refer to donor and standard, respectively. $[Ru(bpy)_3]^{2+}$ was employed as the standard (Φ_{std} = 0.018 ± 0.002 and 0.040 ± 0.002 at λ_{ex} = 450nm in acetonitrile and water, respectively[10]). DNA bound quantum yields for **1-4** were obtained in 5 × 10^{-5} M calf-thymus DNA solution in 5 mM Tris.HCl, 200 mM NaCl, pH 7.5.

$$\Phi_D = \Phi_{std}\left(\frac{m_{dD}}{m_{std}}\right)\left(\frac{\eta_D^2}{\eta_{std}^2}\right) \quad \textit{Equation 2}$$

FRET-related parameters

FRET properties were determined as set out by Algar et al.[11] Spectral overlap, J, was determined employing the normalized fluorescence emission spectrum of Cy5.5 and absorption spectrum of **1-4** scaled by peak molar absorption coefficient, Microsoft Excel was used to calculate the spectral overlap. Förster distance or radius, R_0, was determined by Equation 3 where J = spectral overlap, κ^2 = orientation factor, Φ_D = donor luminescence quantum yield, n = refractive index of the medium.

$$R_0^6 = 0.021 J\kappa^2\Phi_D n^{-4} \quad \textit{Equation 3}$$

Donor-acceptor proximity, r, was calculated by Equation 4, where E_{FRET} = FRET efficiency. E_{FRET} was calculated by Equation 5, where I_{DA} = donor intensity in the presence of the acceptor and I_D = donor emission intensity in the absence of the acceptor.

$$E_{FRET} = \frac{1}{\left(1+\left(\frac{r}{R_0}\right)^6\right)} \quad \textit{Equation 4}$$

$$E_{FRET} = 1 - \frac{I_{DA}}{I_D} \quad \textit{Equation 5}$$

FRET DNA binding assay

In a typical experiment, a concentration gradient of each compound (0.1 - 20 μM) was treated with 1 μM of each DNA structure/sequence in 96 well plates (black, optical bottom, Thermo). 2x concentrations were prepared in advance and mixed 1:1 in each well to achieve the desired concentrations. After 30 mins, fluorescence spectra (λ_{em} = 550-850 nm) or intensity at a fixed wavelength (710 nm for FRET, 630 nm or 605 for MLCT emission of **1**-**4**) were recorded by Tecan Infinite M Nano plate reader (λ_{ex} = 450 nm). The intensity of the FRET emission peak for each compound concentration was measured, background subtracted and normalized to the maximum FRET intensity. Binding curves were generated, fit to a sigmoidal binding model (Origin) and K_d values extrapolated as the concentration required for 50 % binding, as described by Jarmoskaite et al.[12] Only fits with R-squared values of 0.9 or greater were used to derive K_d values, otherwise it was concluded that binding saturation had not been reached and the K_d value was greater than the maximum concentration employed.

Cell culture

MDA-MB-231, HCC38, HCT116 and T24 cell lines were cultured in DMEM supplemented with 10% fetal bovine serum (FBS) and 1% penicillin/streptomycin antibiotic. MCF10A normal breast cell line was cultured in DMEM supplemented with 5% horse serum, 0.5 μg/mL hydrocortisone, 20 ng/mL recombinant human EGF (hEGF), 10 μg/mL insulin and 1 % penicillin/streptomycin antibiotic. Cells were maintained at 37°C under a humified atmosphere containing 5% CO_2 and routinely subcultured with Trypsin.

Cytotoxicity and Olaparib synergy

Cells were seeded in 96 well plates and allowed to adhere for 24 h. Cells were then treated with a concentration gradient of each compound alone or in combination with Olaparib (10 μM). After 72 h, solutions were removed and thiazolyl blue tetrazolium bromide (MTT) reagent dissolved in PBS was added (0.5 mg/mL). After 4 h, the solution was removed and the purple formazan crystals were then solubilized with 100 μL of DMSO and the absorbance at 570 nm (620 nm as reference wavelength) was measured using microplate reader. The average in percent reduction of cell viability was expressed relative to untreated control cells. Half inhibitory IC50 values were determined using GraphPad Prism software. Combination indices (CI) were calculated using CalcuSyn and CompuSyn software (Biosoft, Cambridge, UK) as established by Chou and Talalay.[13] CI < 1.0 indicates synergism, CI = 1.0 indicates additive, and CI > 1 indicates antagonism. GraphPad Prism Software was used to generate a 3-color

scale based on CI values obtained, where synergism is represented by green, additive by yellow, and antagonism by red. The colors of each CI value were interpolated in between these constraints accordingly.

DNA fiber assay

MDA-MB-231 cells were treated with 20 μM **3** or **4** for 1 h, complexes were removed, and pulse-labeled with 25 μM CldU (Sigma-Aldrich) and 250 μM IdU (Sigma-Aldrich) for 20 min each. The DNA fiber assay, including image acquisition and data processing, was then performed as described within a recent publication.[14]

Cell-cycle analysis

Cells were seeded at 3 x 10^5 cells/well 6-well plates and allowed to adhere for 24 h. Cells were then treated as stated in the main text, trypsinized and washed with PBS twice. Cells were then fixed with ice-cold 70% ethanol, centrifuged at 1,000 rpm for 5 min, and the resulting cell pellets were washed with PBS twice. Samples were resuspended in 500 μL PBS and treated with 5 μL of RNase A solution (10 mg/mL). After 15 min of incubation, the samples were stained with 2 μL propidium iodide (PI) (5 mg/mL) in the dark at room temperature. Samples were acquired and analysed with a NovoCyte flow cytometer (Agilent Technologies) and NovoExpress software. For each sample, a minimum of 10,000 cells were counted.

Annexin V binding assay

Cells were seeded at 3 x 10^5 cells/well 6-well plates and allowed to adhere for 24 h. Cells were then treated as stated in the main text, trypsinized and washed with PBS twice. Then, 500 μL 1X binding buffer and 5 μL Annexin V-FITC (Invitrogen) was added for 20 min at RT. 5 μL PI (20 μg/mL) was added prior to flow cytometric analysis using a NovoCyte flow cytometer and the results were analysed using NovoExpress software. For each sample, a minimum of 10,000 cells were counted.

Supplementary Tables

Table S1 Photophysical properties of 3 and 4 (as PF_6 salts) in acetonitrile.

Complex	λ_{abs} (ε / 10^4 M^{-1} cm^{-1}) / nm	λ_{em} (λ_{ex} / nm) / nm	Φ
**3**	460 (1.3), 426 (1.6), 366 (2.7), 457 (2.8), 314 (3.5), 279 (12.3), 204 (7.5)	606 (460)	0.0236
**4**	509 (1.1), 473 (1.7), 424 (1.6), 343 (6.6), 336 (6.8), 298 (12.9), 265 (5.0), 251 (4.8), 217 (6.7)	609 (473)	0.0021

Table S2 Luminescent lifetimes of unlabeled or Cy5.5-labeled 20mer duplex DNA with addition of 1.

	Unlabeled DNA + **1**	Cy5.5-labeled DNA	Cy5.5-labeled DNA + **1** (1:1)	Cy5.5-labeled DNA + **1** (1:10)
λ_{ex}(nm)/λ_{em} (nm)	450/630	683/705	450/705	450/705
Average lifetime (ns)	94.41	1.18	2.36	3.62
Fit	Bi-exponential	Single exponential	Bi-exponential	Bi-exponential

Table S3. Selectivity indices (SI) of cisplatin, 2, 3 or 4 towards MDA-MB-231 or HCC38 breast cancer, HCT116 colorectal carcinoma, T24 bladder cancer cell lines as compared to MCF10A epithelial cells. SI = IC_{50} MCF10A/IC_{50} cancer cell line. ND = not determined.

Complex	MDA-MB-231	HCC38	HCT116	T24
Cisplatin	0.8	5.9	5.3	17.7
**2**	ND	ND	>1.9	>2.9
**3**	>4.6	>26.3	>6.1	>3.6
**4**	3.1	3.9	6.4	2.1

Table S4. Half-inhibitory (IC_{50}) values of 2, 3 or 4 in combination with 10 μM Olaparib in MDA-MB-231 or HCC38 breast cancer, HCT116 colorectal carcinoma, T24 bladder cancer or the MCF10A non-tumorigenic epithelial cell line (72 h treatment). Data are mean ± SD of three independent experiments. ND = not determined due to increased Olaparib single-agent cytotoxicity.

Complex/combination	MDA-MB-231	HCC38	HCT116	T24	MCF10A
**2** + OLP	32.9 ± 7.75	ND	ND	17.0 ± 2.3	78.3 ± 14.1
**3** + OLP	6.8 ± 4.1	ND	ND	15.3 ± 2.5	>100
**4** + OLP	7.7 ± 8.4	ND	ND	12.9 ± 0.9	38.7 ± 3.4

Supplementary Figures

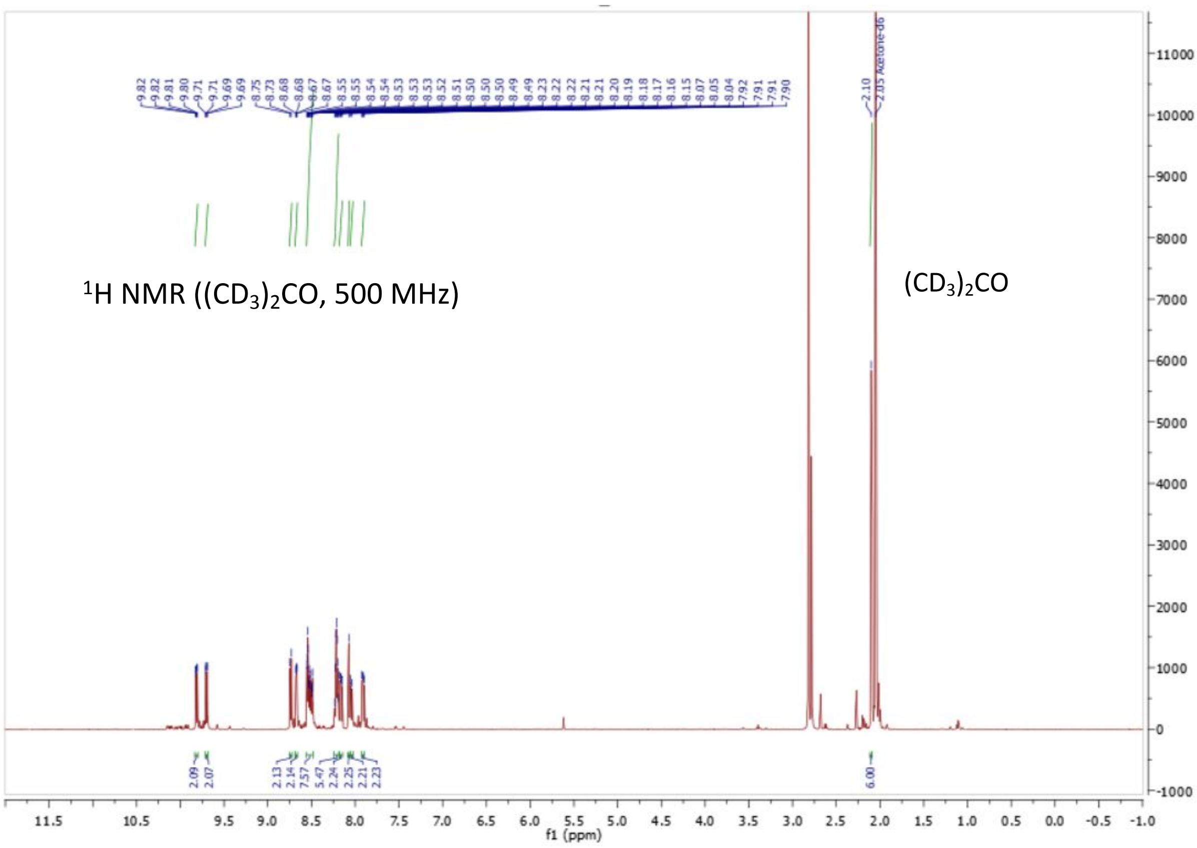

Figure S1. ^{1}H NMR (500 MHz, $(CD_3)_2CO$) spectrum of **3**.

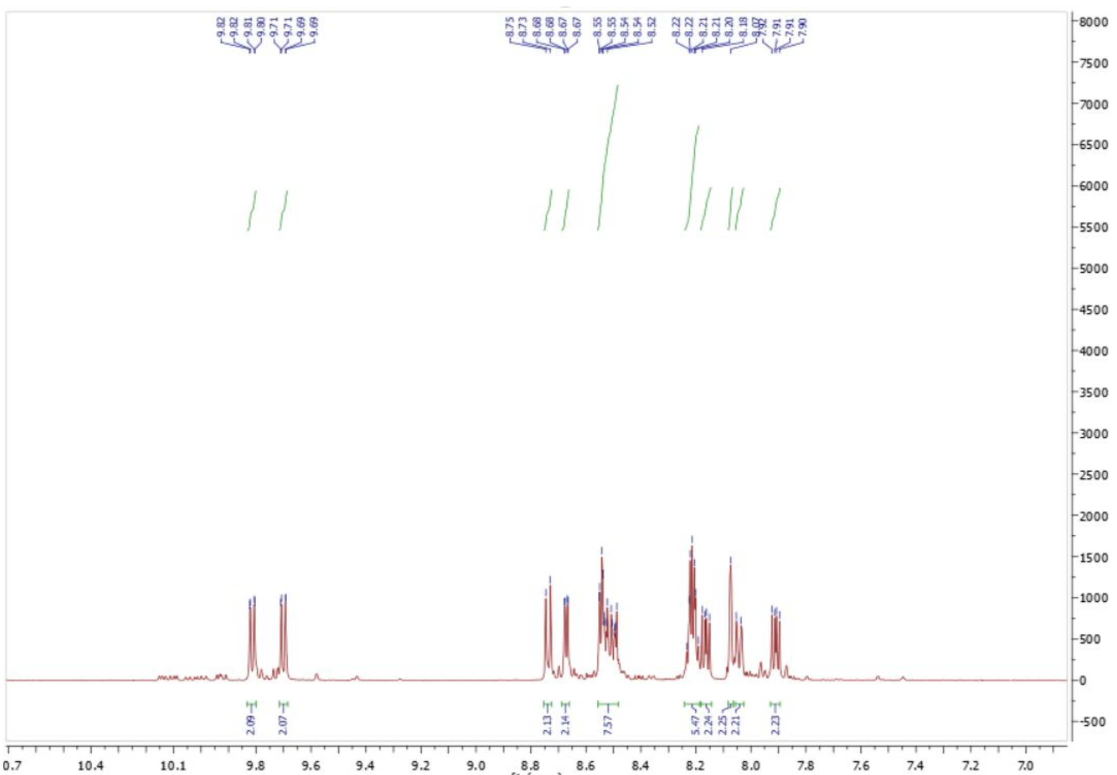

Figure S2. Aromatic region of ^{1}H NMR (500 MHz, $(CD_3)_2CO$) spectrum of **3**.

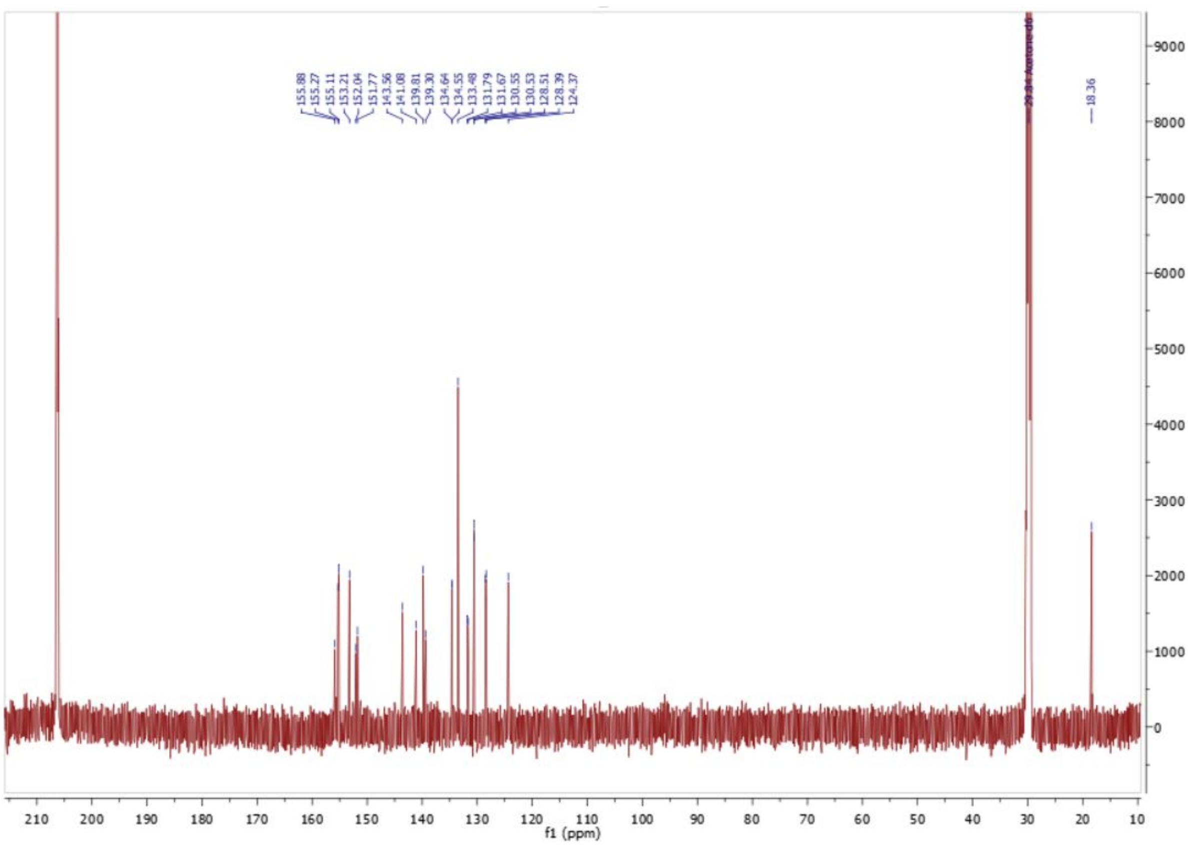

Figure S3. ^{13}C NMR (126 MHz, $(CD_3)_2CO$) spectrum of **3**.

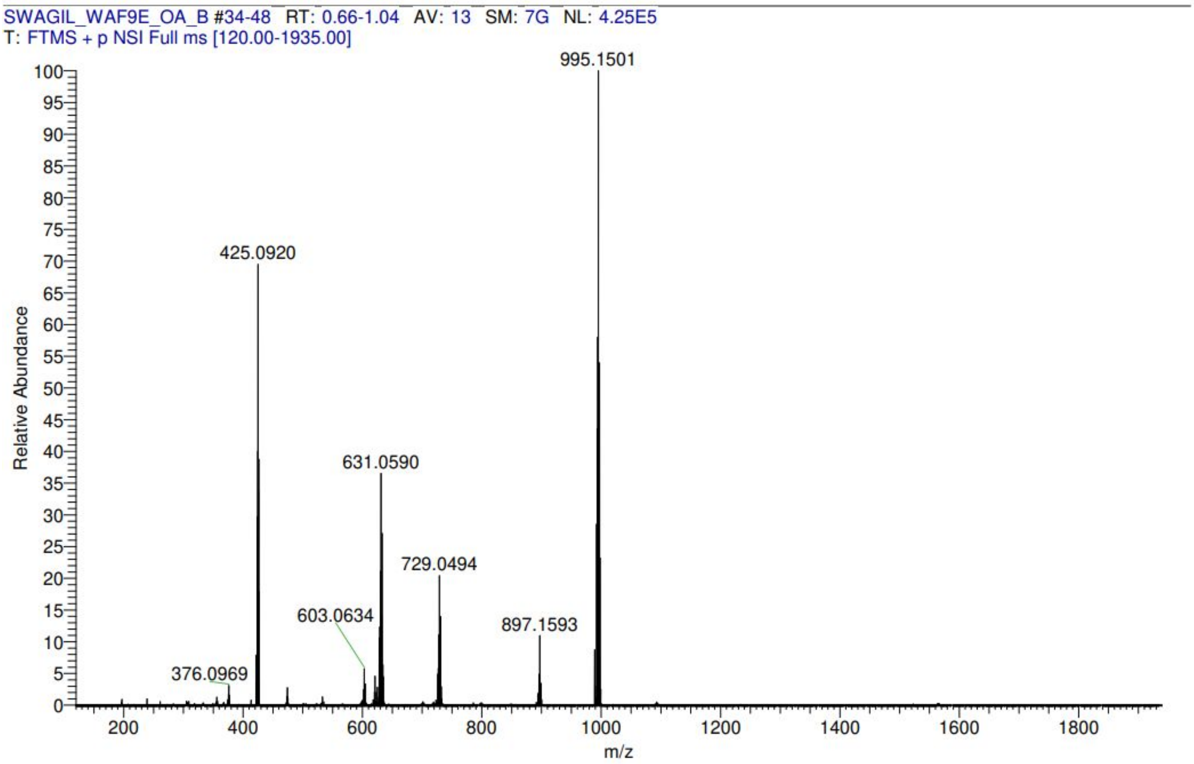

Figure S4. ESI-MS spectrum of **3**.

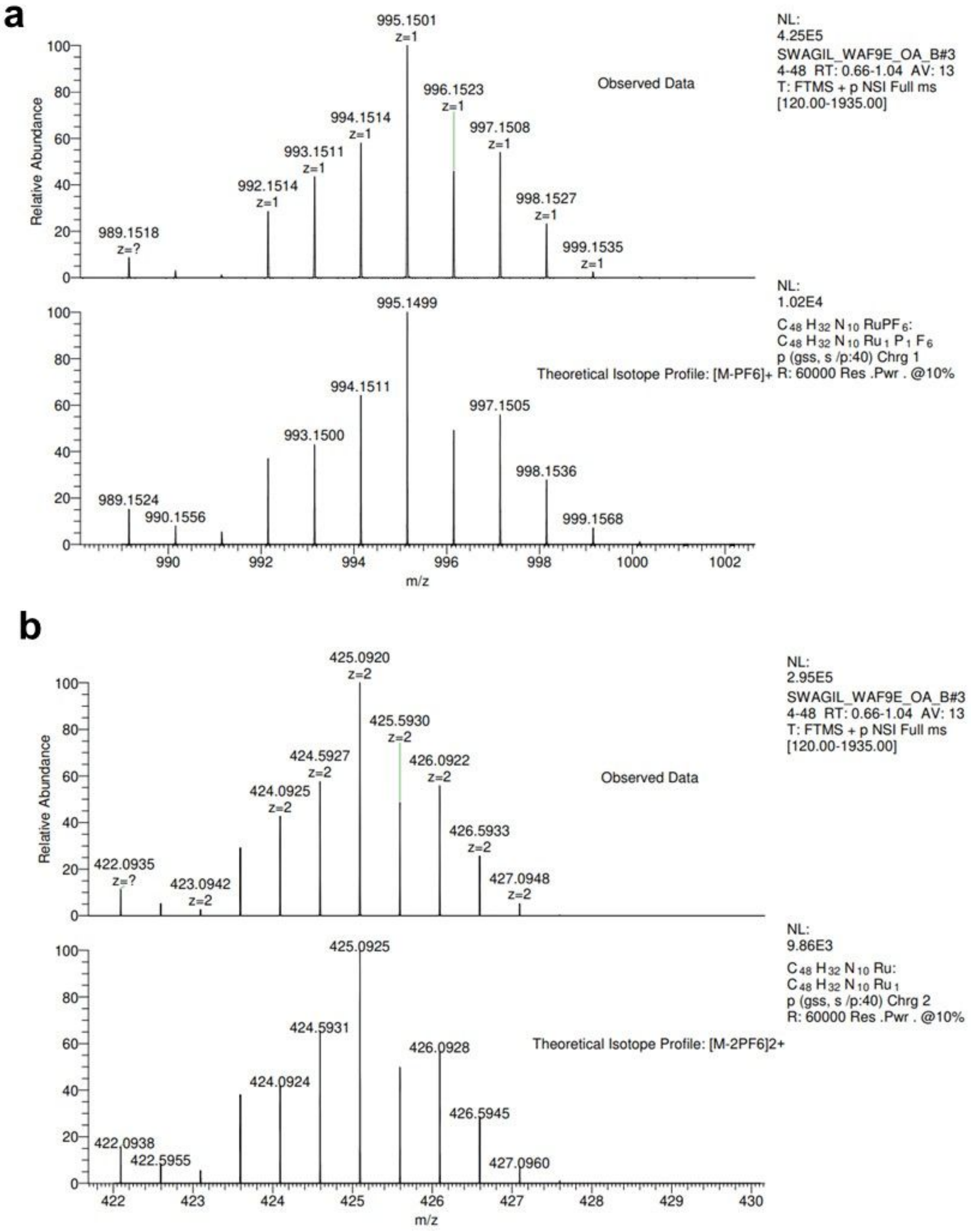

Figure S5. Observed and theoretical isotope ratio of $[M - PF_6]^+$ (a) and $[M - 2PF_6]^{2+}$ (b) peaks from ESI-MS spectrum of **3**.

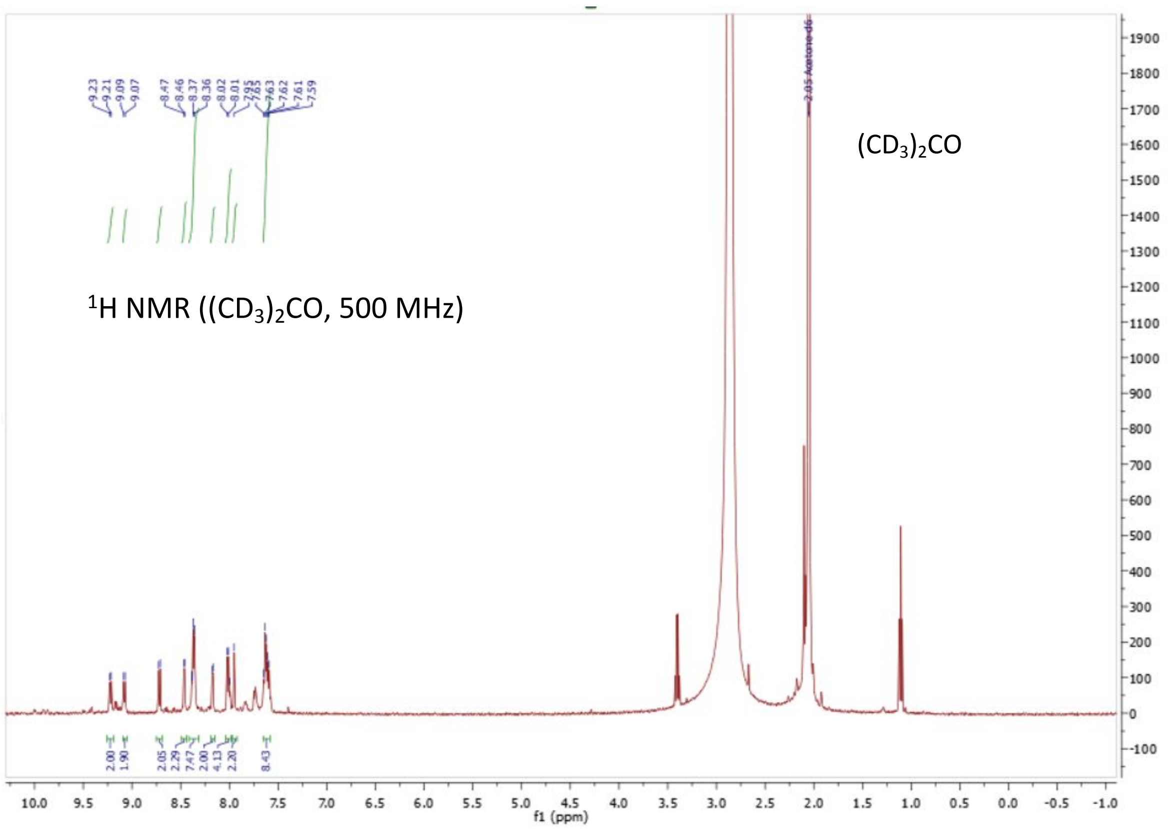

Figure S6. ^{1}H NMR (500 MHz, $(CD_3)_2CO$) spectrum of **4**.

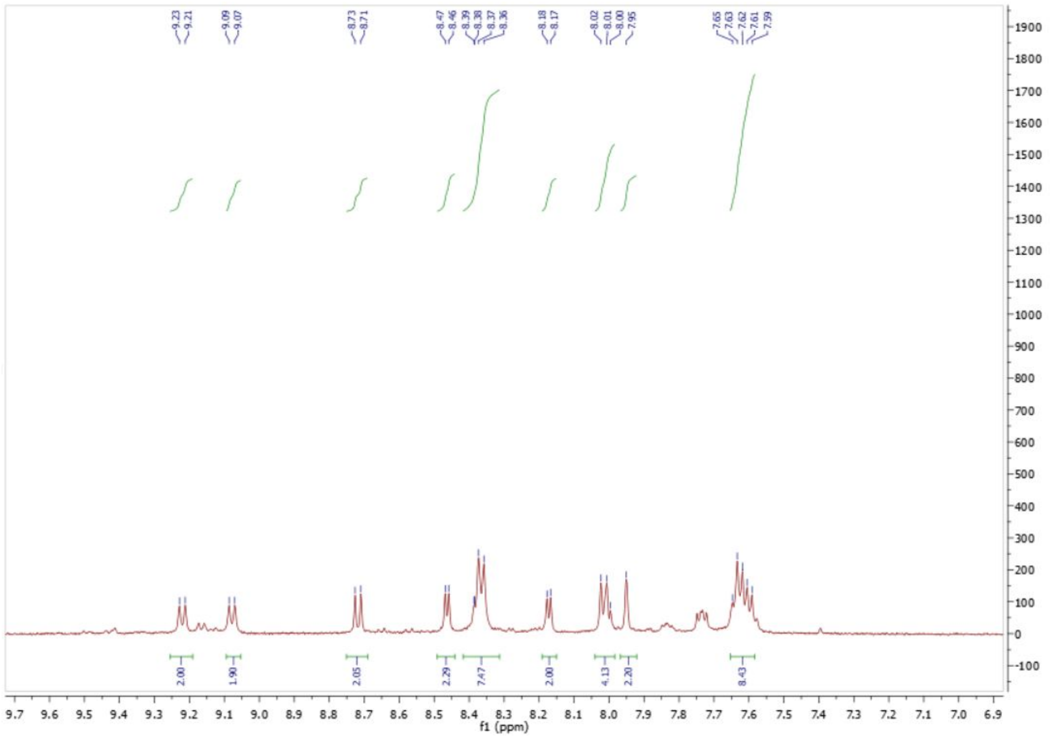

Figure S7. Aromatic region of ^{1}H NMR (500 MHz, $(CD_3)_2CO$) spectrum of **4**.

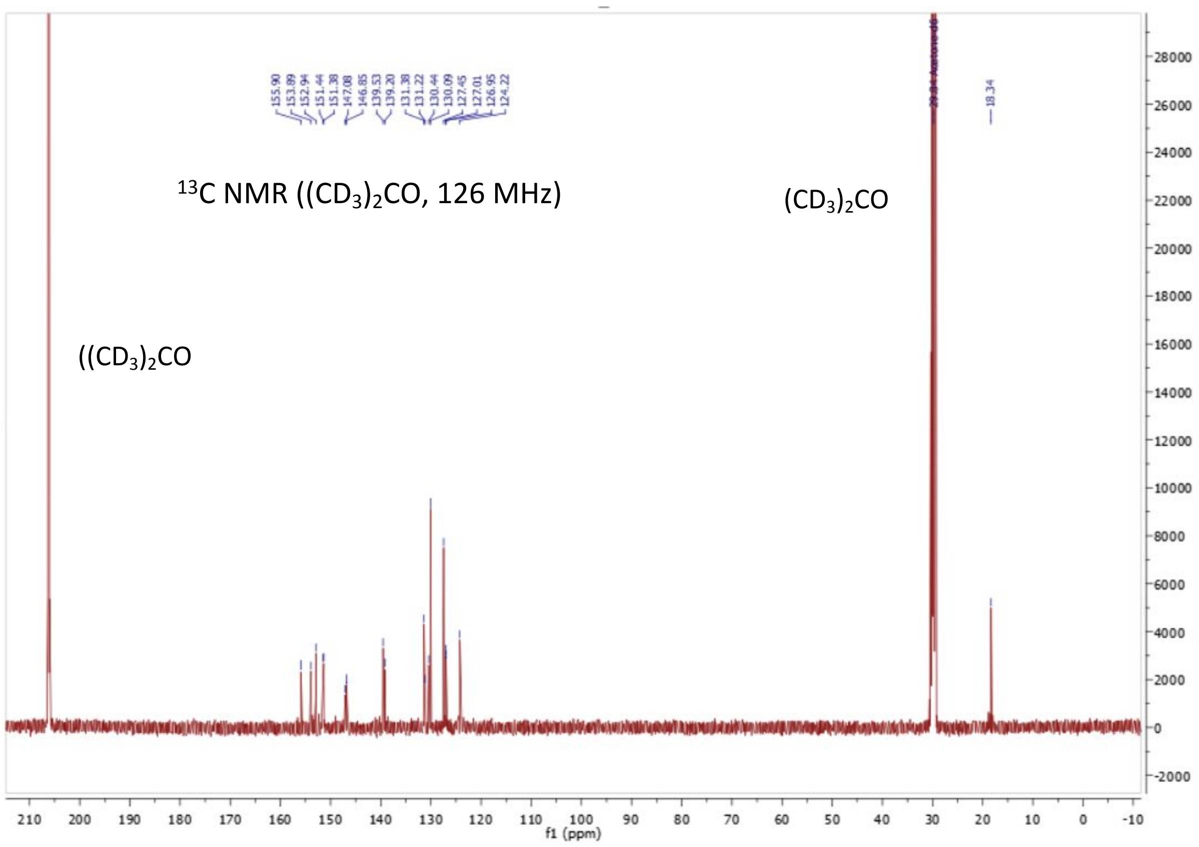

Figure S8. ^{13}C NMR (126 MHz, $(CD_3)_2CO$) spectrum of **4**.

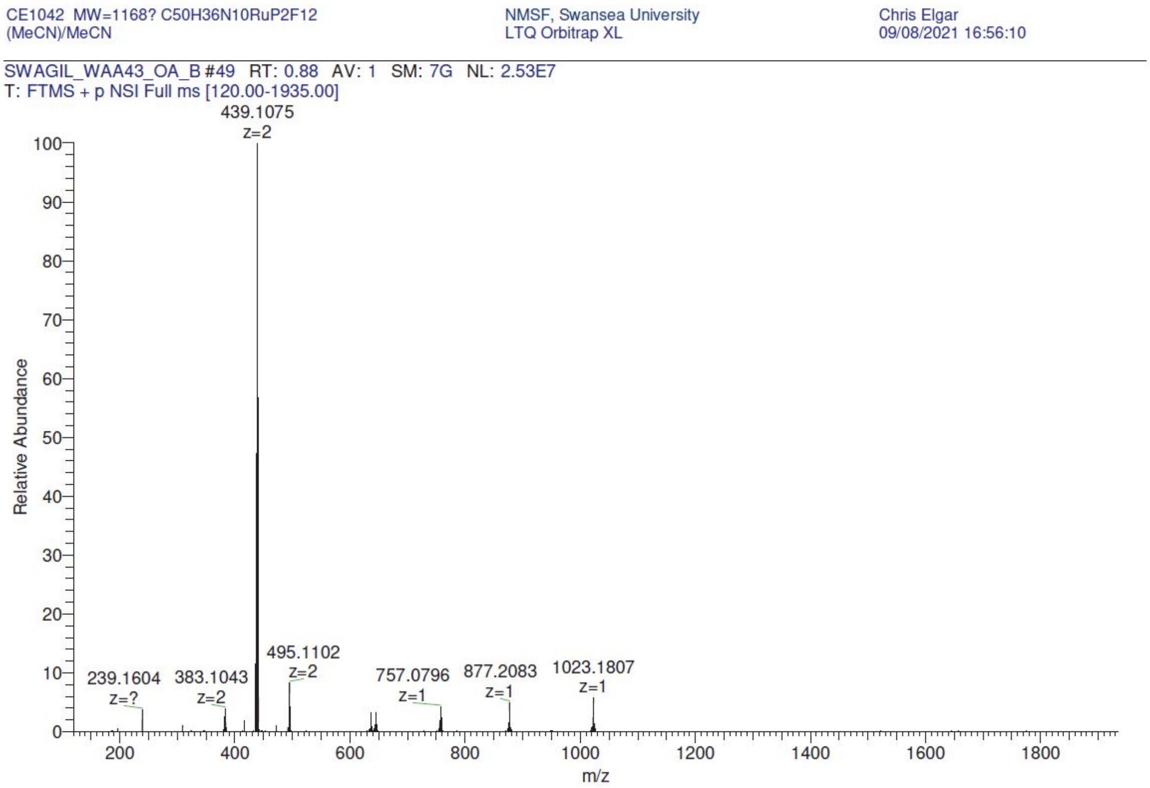

Figure S9. ESI-MS spectrum of **4**.

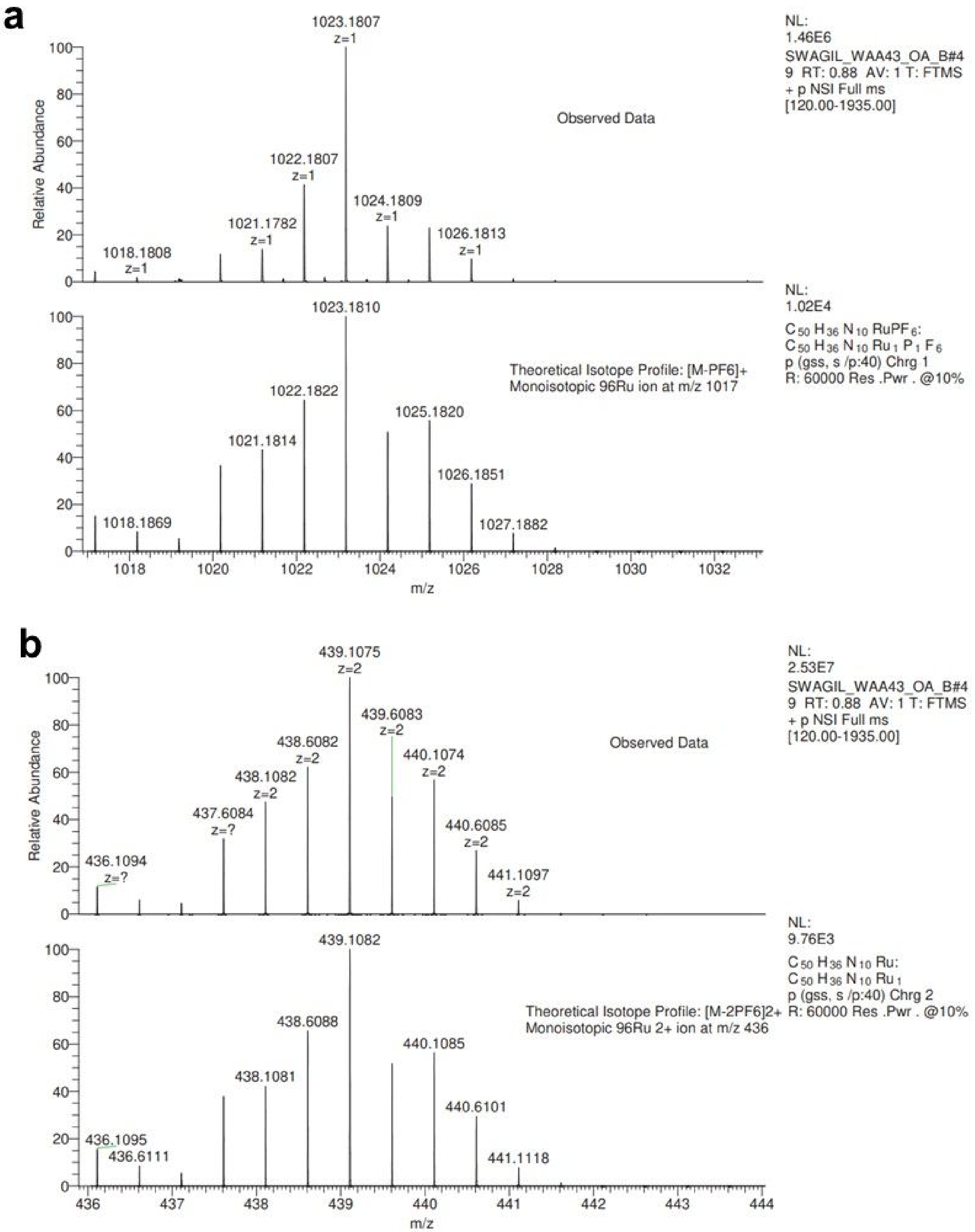

Figure S10. Observed and theoretical isotope ratio of $[M - PF_6]^+$ (a) and $[M - 2PF_6]^{2+}$ (b) peaks from ESI-MS spectrum of **4**.

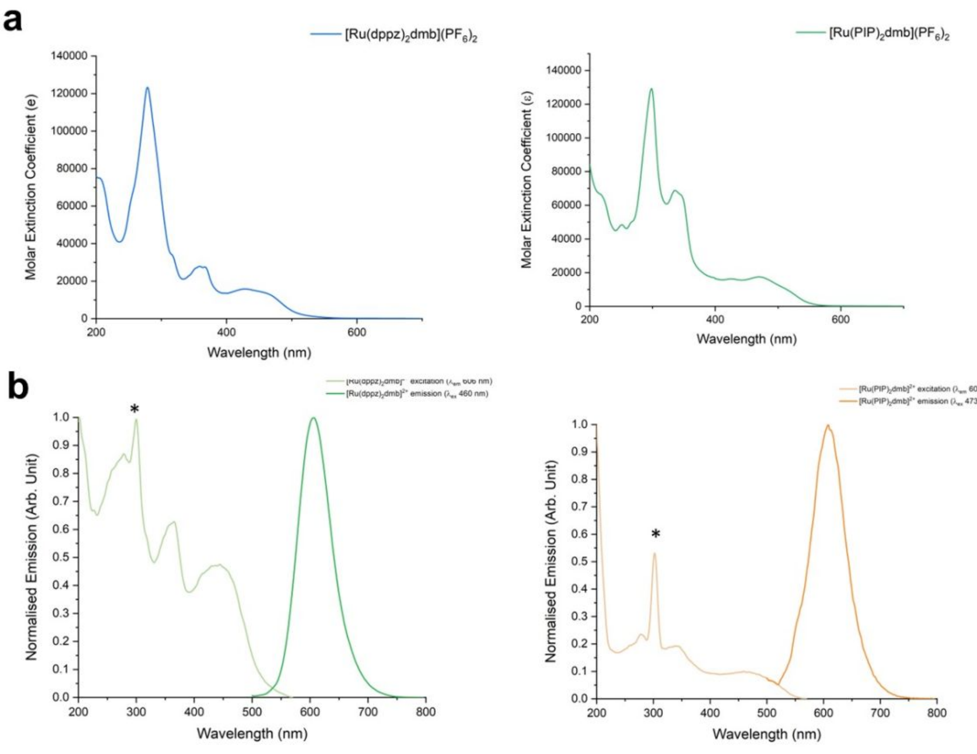

Figure S11. (a) UV-vis absorbance spectra of **3** (left) and **4** (right) in acetonitrile. (b) Emission and excitation spectra for **3** (left) and **4** (right) in acetonitrile. *Represents lamp harmonics in the excitation spectra.

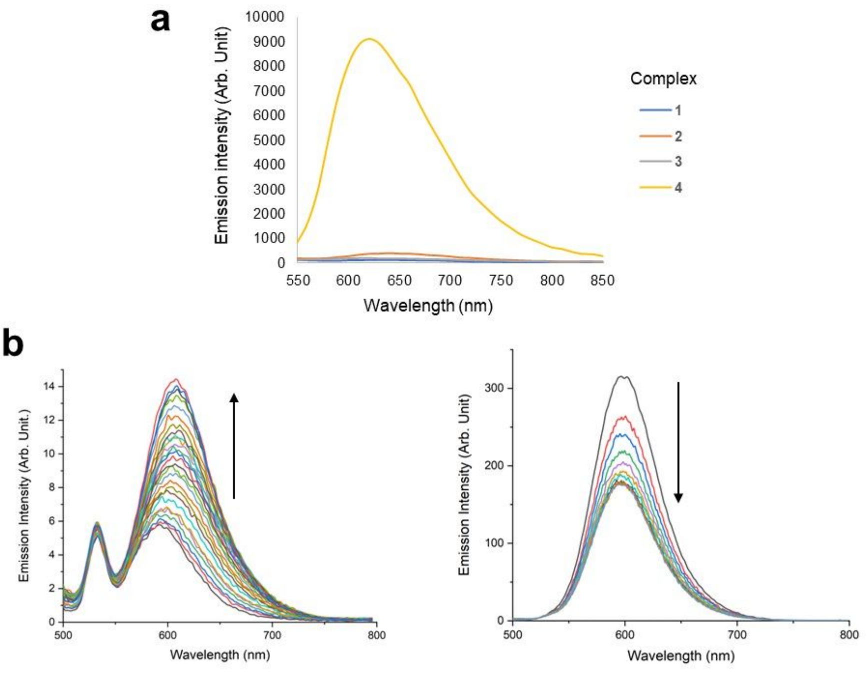

Figure S12. (a) Relative emission of **1-4** (20 μM) in TRIS buffer. (b) Addition of calf thymus DNA to solutions of **3** (left) or **4** (right).

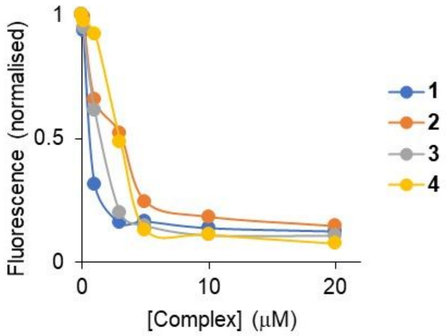

	K_{app} (× 10^7 M^{-1})
**1**	13.7
**2**	5.7
**3**	2.8
**4**	2.8

Figure S13. Ethidium bromide displacement assay and derived K_{app} binding constants for **1-4**.

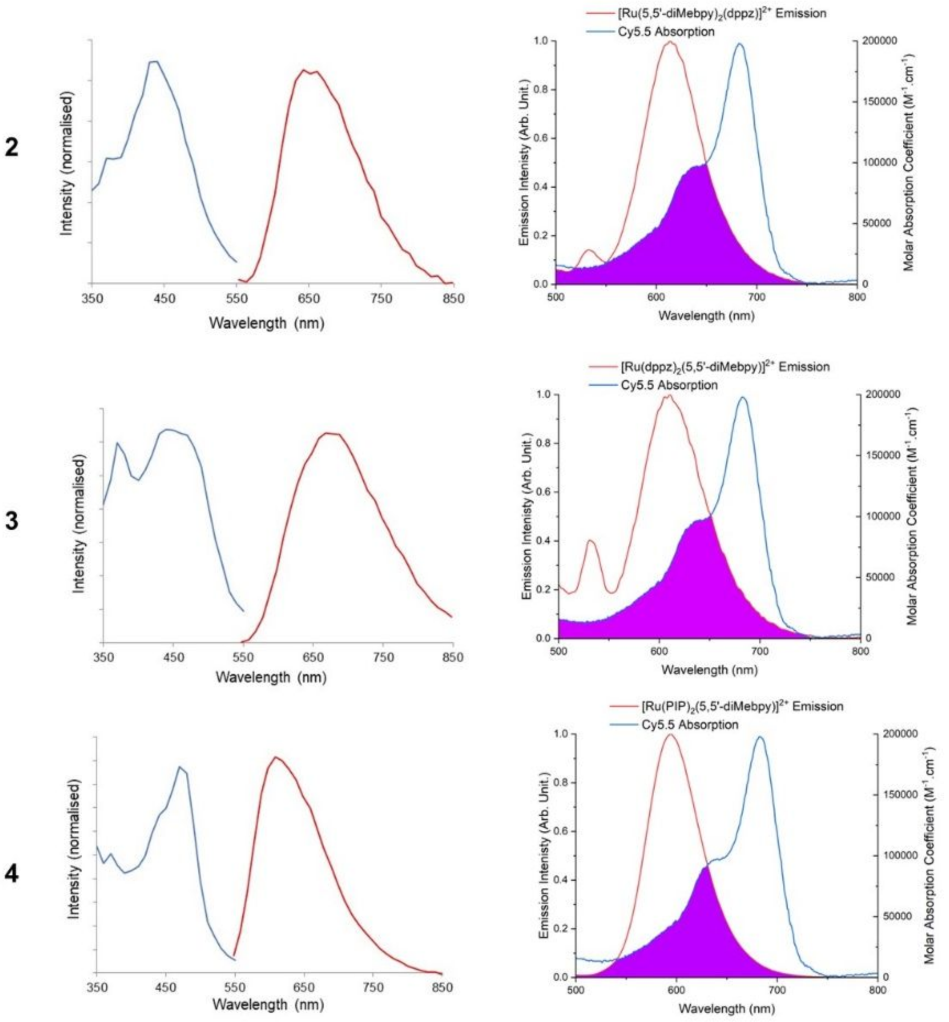

Figure S14. Left, excitation and emission spectra of **2-4** with unlabeled 20mer DNA. Right, Spectral overlap of DNA bound **2-4** (from unlabeled duplex DNA) and Cy5.5 absorption.

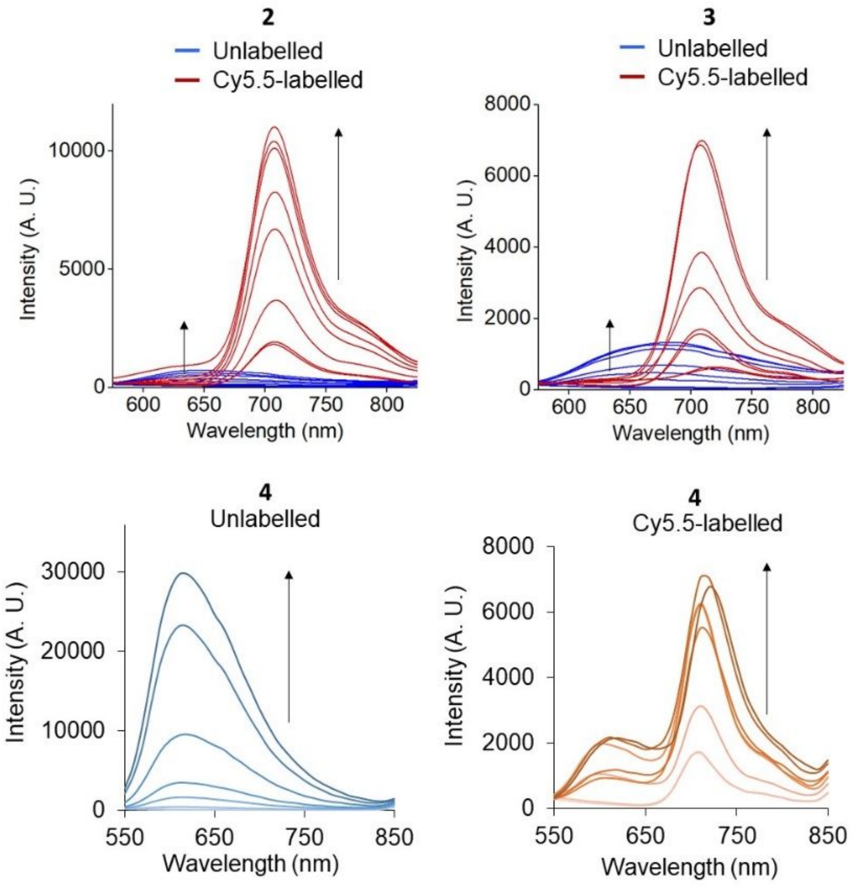

Figure S15. Red, emission spectra (λ_{ex} = 450 nm) of Cy5.5-labeled 20 mer (1 μM) with increasing concentration of **2**, **3** or **4** (0.1-20 μM). Blue, addition of **2**, **3** or **4** to unlabeled DNA showing MLCT emission (λ_{ex} = 450 nm).

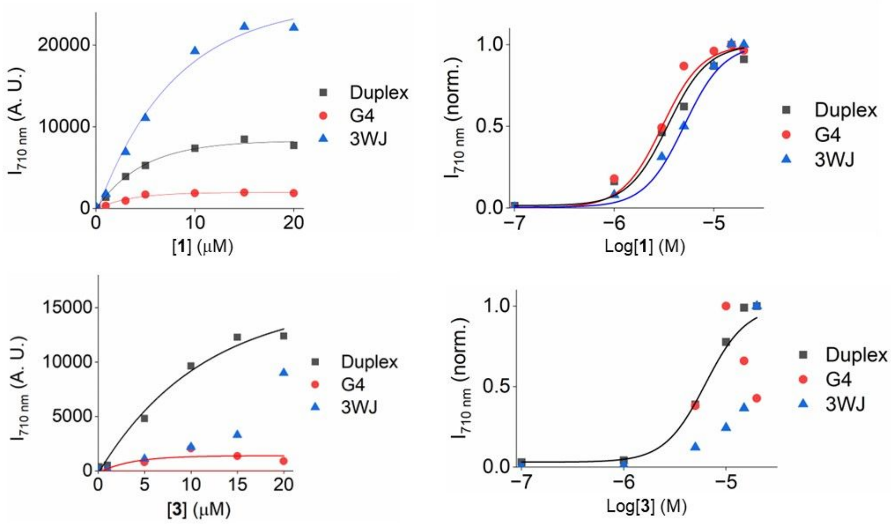

Figure S16. FRET intensity (left) and dose-response curve (right) generated from addition of **1** or **3** (0.1 – 20 μM) to Cy5.5-labeled duplex, G4 or 3WJ DNA (1 μM). λ_{ex} = 450 nm, λ_{em} = 710 nm. FRET intensity was background corrected. 3WJ and duplex buffer: 5 mM Tris, 200 mM NaCl, pH 7.5. G4 buffer: 100 mM KCl, 10 mM Tris.HCl, pH 7.4.

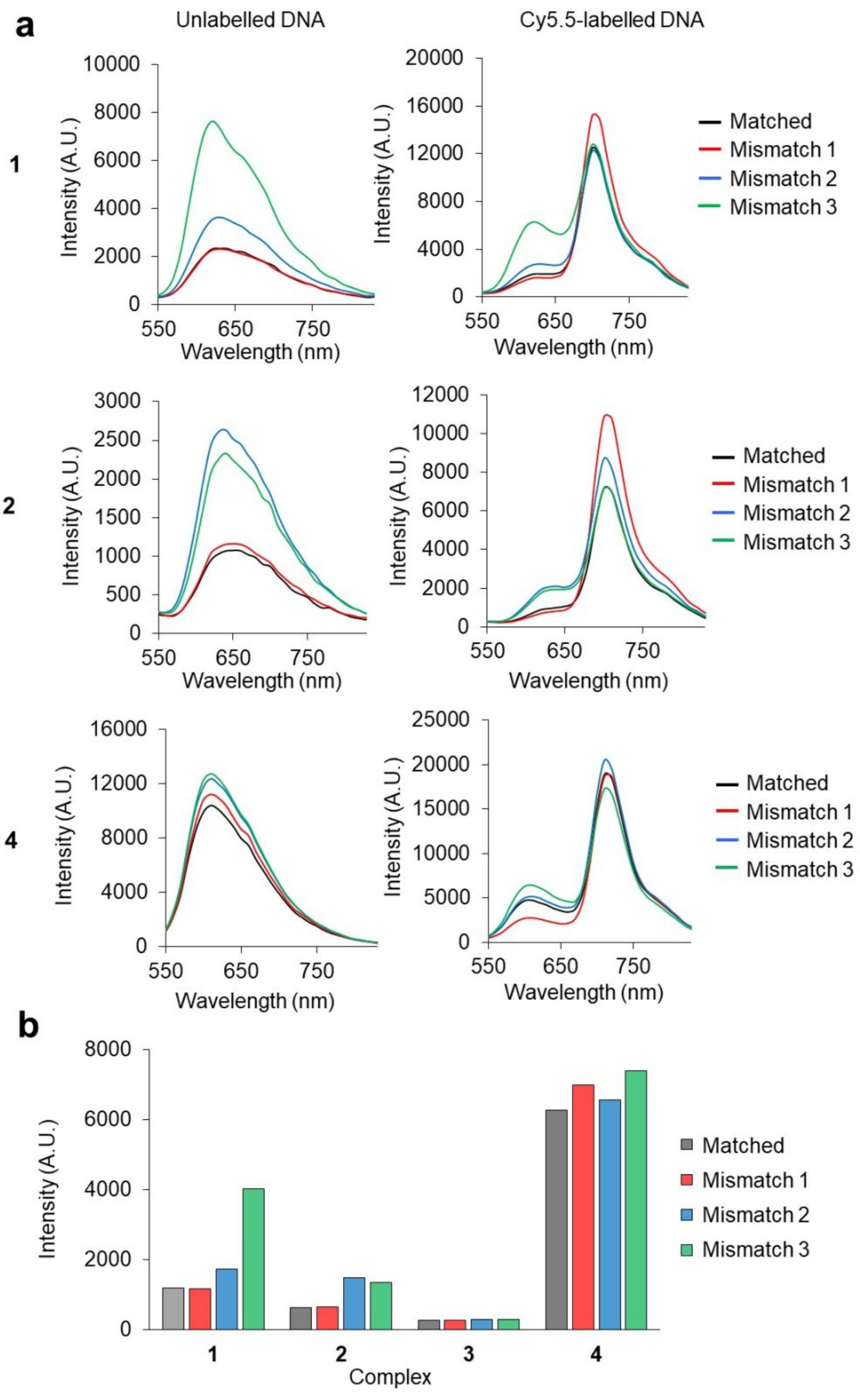

Figure S17. (a) Emission spectra of **1**, **2** or **4** (5 μM) and unlabeled (left) or Cy5.5-labeled (right) 27-mers. 1 μM DNA employed in each case. λ_{ex} = 450 nm, all emission spectra were collected using the same optical parameters. (b) Maximum MLCT intensity of **1-4** (3 μM) with addition of unlabeled 27mer duplexes (1 μM). λ_{ex} = 450 nm. Buffer: 5 mM Tris, 200 mM NaCl, pH 7.5.

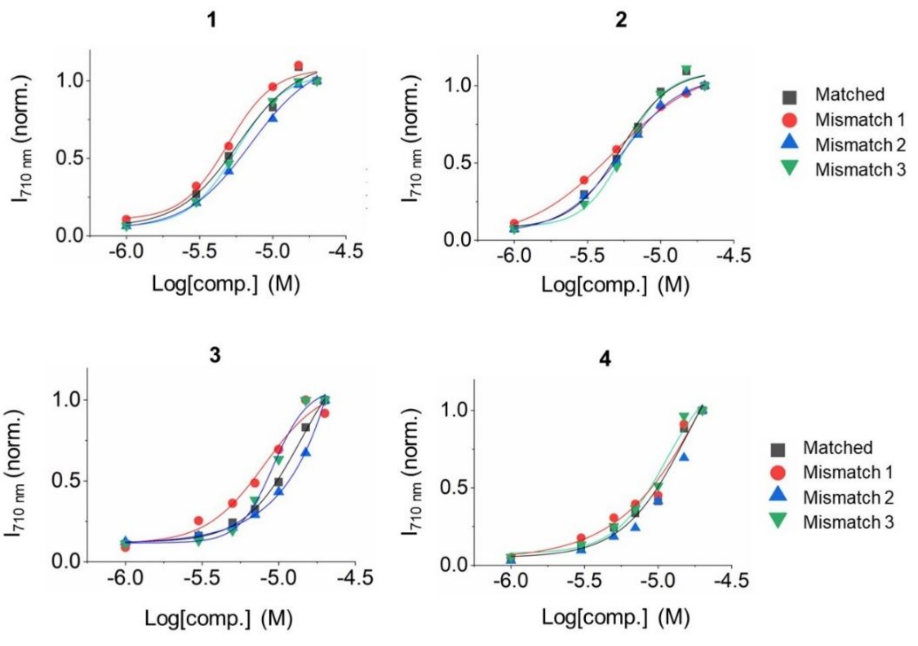

Figure S18. Representative dose-response curves generated from addition of **1-4** to Cy5.5-labeled 27mers. λ_{ex} = 450 nm, λ_{em} = 710 nm. FRET intensity was background corrected. Buffer: 5 mM Tris, 200 mM NaCl, pH 7.5.

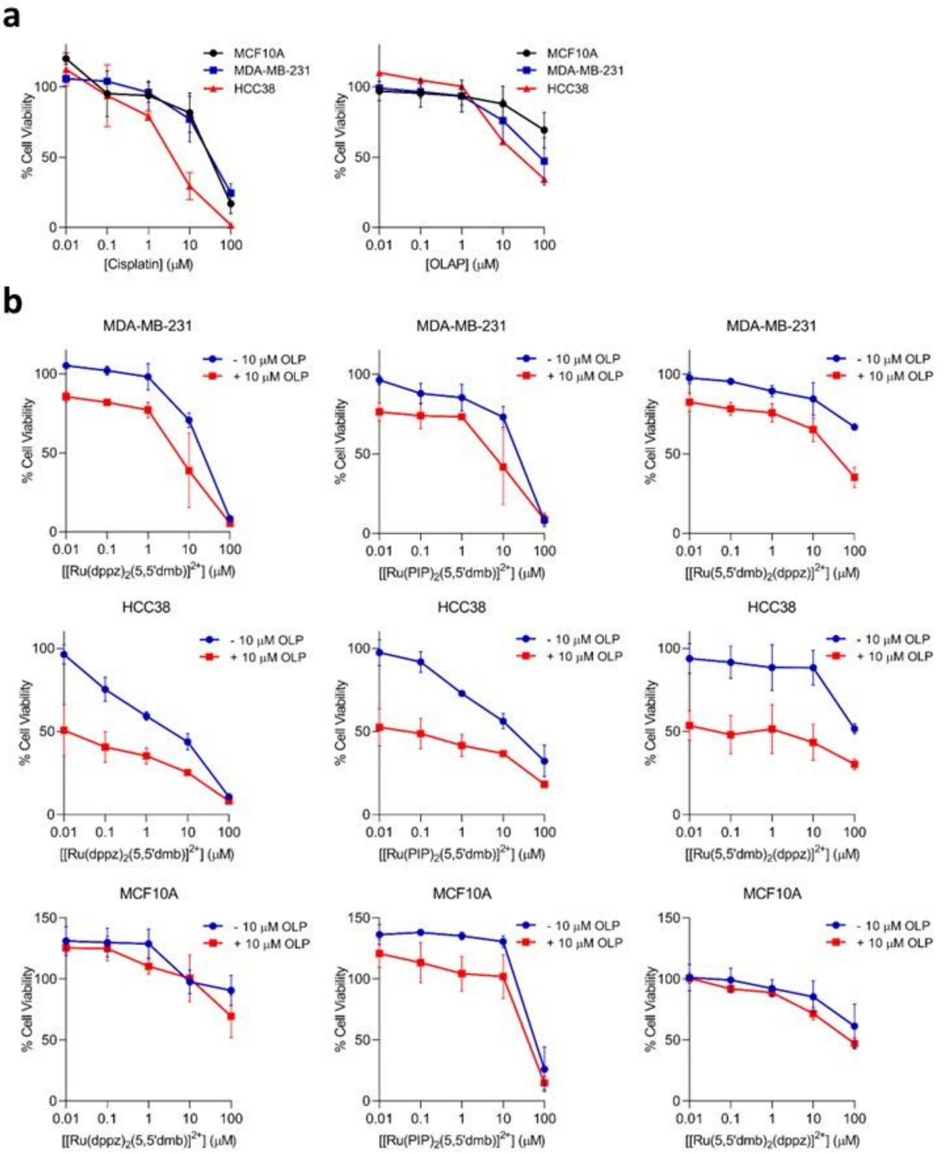

Figure S19. (a) Cell viability of HCC38, MDA-MB-231 or MCF10A cells upon treatment with concentration gradient of cisplatin (left) and Olaparib (OLP, right). MCF10A and MDA-MB-231 data for Olaparib from Yusoh et al.[15] (b) Cell viability of cells upon treatment with compounds alone or in combination with 10 µM Olaparib for 72 h, as determined by MTT assay. Data mean ± SD of three independent experiments (n = 3).

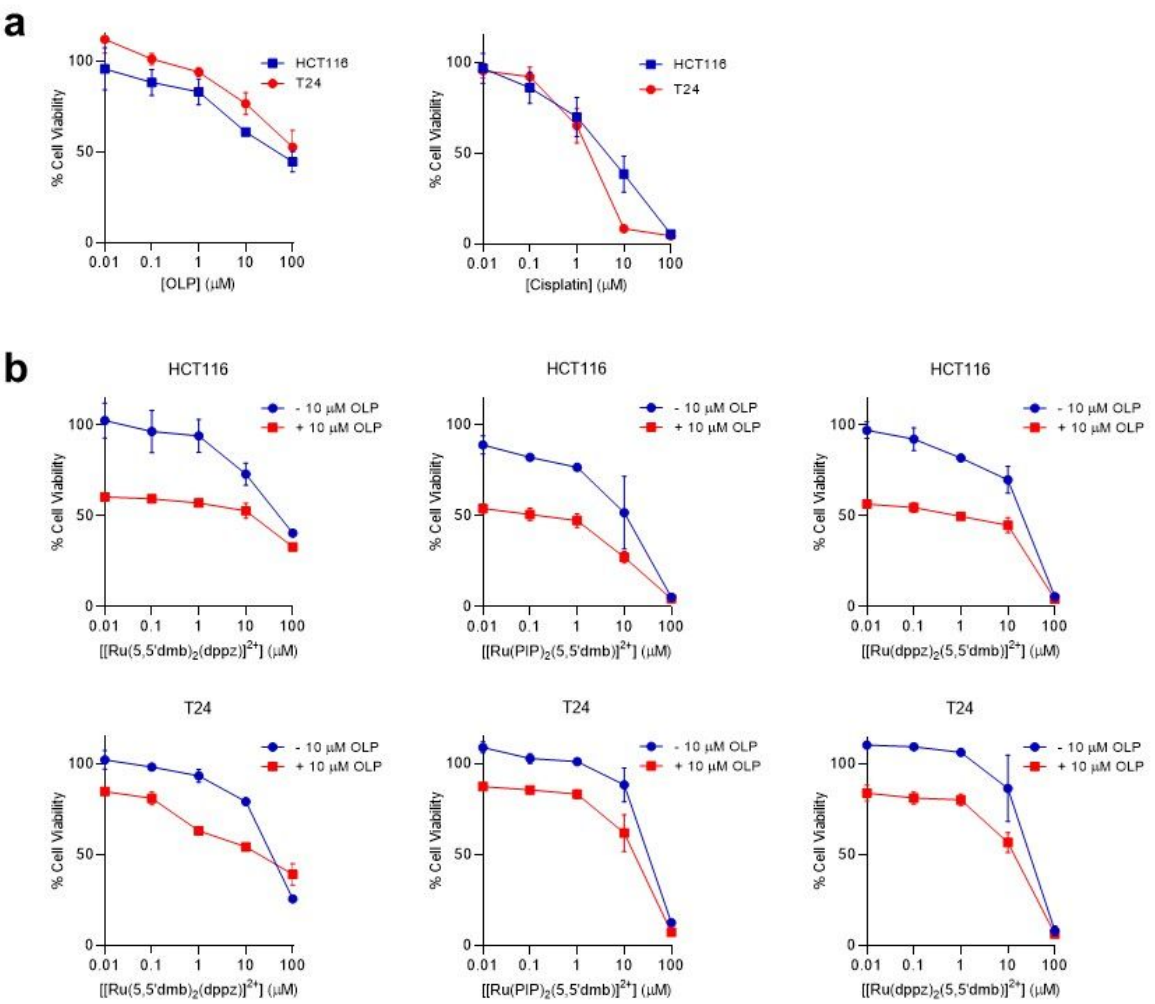

Figure S20. (a) Cell viability of HCT116 or T24 cells upon treatment with concentration gradient of Olaparib (OLP, left) or cisplatin (right). (b) Cell viability of cells upon treatment with compounds alone or in combination with 10 μM Olaparib for 72 h, as determined by MTT assay. Data mean ± SD of three independent experiments (n = 3).

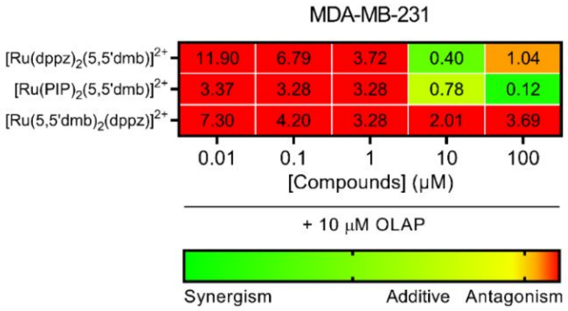

Figure S21. Combination indices (CIs) for various Olaparib combinations in MDA-MB-231 cells for 72 h treatment. CI values were calculated using CompuSyn software, and a heat map was generated as described within Experimental section.

References

(1) Sullivan, B. P.; Salmon, D. J.; Meyer, T. J. Mixed Phosphine 2,2'-Bipyridine Complexes of Ruthenium. *Inorg. Chem.* **1978**, *17* (12), 3334–3341.

(2) Friedman, A. E.; Chambron, J. C.; Sauvage, J. P.; Turro, N. J.; Barton, J. K. A Molecular Light Switch for DNA: Ru(Bpy)2(Dppz)2+. *J. Am. Chem. Soc.* **1990**, *112* (12), 4960–4962.

(3) Boynton, A. N.; Marcélis, L.; Barton, J. K. [Ru(Me4phen)2dppz]2+, a Light Switch for DNA Mismatches. *J. Am. Chem. Soc.* **2016**, *138* (15), 5020–5023.

(4) McGhee, J. D.; von Hippel, P. H. Theoretical Aspects of DNA-Protein Interactions: Co-Operative and Non-Co-Operative Binding of Large Ligands to a One-Dimensional Homogeneous Lattice. *J. Mol. Biol.* **1974**, *86* (2), 469–489.

(5) Gill, M. R.; Cecchin, D.; Walker, M. G.; Mulla, R. S.; Battaglia, G.; Smythe, C.; Thomas, J. A. Targeting the Endoplasmic Reticulum with a Membrane-Interactive Luminescent Ruthenium(Ii) Polypyridyl Complex. *Chem. Sci.* **2013**, *4* (12), 4512–4519.

(6) Gill, M. R.; Walker, M. G.; Able, S.; Tietz, O.; Lakshminarayanan, A.; Anderson, R.; Chalk, R.; El-Sagheer, A. H.; Brown, T.; Thomas, J. A.; Vallis, K. A. An 111In-Labeled Bis-Ruthenium(Ii) Dipyridophenazine Theranostic Complex: Mismatch DNA Binding and Selective Radiotoxicity towards MMR-Deficient Cancer Cells. *Chem. Sci.* **2020**, *11* (33), 8936–8944.

(7) McCann, M.; McGinley, J.; Ni, K.; O'Connor, M.; Kavanagh, K.; McKee, V.; Colleran, J.; Devereux, M.; Gathergood, N.; Barron, N.; Prisecaru, A.; Kellett, A. A New Phenanthroline–Oxazine Ligand: Synthesis, Coordination Chemistry and Atypical DNA Binding Interaction.

Chem. Commun. **2013**, *49* (23), 2341–2343.

(8) Waller, Z. A. E.; Sewitz, S. A.; Hsu, S.-T. D.; Balasubramanian, S. A Small Molecule That Disrupts G-Quadruplex DNA Structure and Enhances Gene Expression. *J. Am. Chem. Soc.* **2009**, *131* (35), 12628–12633.

(9) Zhu, J.; Haynes, C. J. E.; Kieffer, M.; Greenfield, J. L.; Greenhalgh, R. D.; Nitschke, J. R.; Keyser, U. F. FeII4L4 Tetrahedron Binds to Nonpaired DNA Bases. *J. Am. Chem. Soc.* **2019**, *141* (29), 11358–11362.

(10) Brouwer, A. M. Standards for Photoluminescence Quantum Yield Measurements in Solution. *Pure App Chem* **2011**, *83* (12), 2213–2228.

(11) Algar, W. R.; Hildebrandt, N.; Vogel, S. S.; Medintz, I. L. FRET as a Biomolecular Research Tool — Understanding Its Potential While Avoiding Pitfalls. *Nat. Methods* **2019**, *16* (9), 815–829.

(12) Jarmoskaite, I.; AlSadhan, I.; Vaidyanathan, P. P.; Herschlag, D. How to Measure and Evaluate Binding Affinities. *Elife* **2020**, *9*, e57264.

(13) Chou, T.-C.; Talalay, P. Quantitative Analysis of Dose-Effect Relationships: The Combined Effects of Multiple Drugs or Enzyme Inhibitors. *Adv. Enzyme Regul.* **1984**, *22*, 27–55.

(14) Bennett, L. G.; Wilkie, A. M.; Antonopoulou, E.; Ceppi, I.; Sanchez, A.; Vernon, E. G.; Gamble, A.; Myers, K. N.; Collis, S. J.; Cejka, P.; Staples, C. J. MRNIP Is a Replication Fork Protection Factor. *Sci. Adv.* **2022**, *6* (28), eaba5974.

(15) Yusoh, N. A.; Tiley, P. R.; Harun, S. N.; Thomas, J. A.; Saad, N.; Chia, S. L.; Gill, M. R.; Ahmad, H. Discovery of Olaparib and Ruthenium(II) Metallocompound Synergy for Cancer Combination Therapy. *under Rev.* **2022**.
